# Exogenous MeJA modulates postharvest tomato aroma by suppressing JAs-ethylene signaling crosstalk

**DOI:** 10.3389/fpls.2025.1712703

**Published:** 2025-12-01

**Authors:** Xuehui Li, Xiaoyu Tan, Yubang Gao, Xinyu Luo, Dongguang Xu, Yiting Wang, Xinli Geng, Xiaolin Yang, Yuhua Xie, Qiuhong Niu, Xiaopu Ren, Libin Wang

**Affiliations:** 1 College of Life Science, Nanyang Normal University, Nanyang, Henan, China; 2 College of Food Science and Technology, Nanjing Agricultural University, Nanjing, Jiangsu, China; 3 School of Agronomy and Horticulture, Jiangsu Vocational College of Agriculture and Forestry, Zhenjiang, Jiangsu, China; 4 National Engineering Research Center for Efficient Utilization of Soil and Fertilizer Resources, College of Resources and Environment, Shandong Agricultural University, Taian, Shandong, China; 5 Research and Development Center for Facility Agriculture and Specialty Agriculture of Xinjiang Uygur Autonomous Region, Shanshan, Xinjiang, China; 6 State Key Laboratory of Efficient Utilization of Agricultural Water Resources, China Agricultural University, Beijing, China; 7 School of Food and Biological Engineering, Hezhou University, Hezhou, Guangxi, China

**Keywords:** tomato fruit, JA-Ile, ethylene, aroma profile, molecular mechanism

## Abstract

**Background:**

Until recently, the mechanism underlying methyl jasmonate (MeJA)-mediated suppression of ethylene metabolism and its impact on quality formation in ripening tomatoes has not been fully clarified. This study aimed to investigate how exogenous MeJA application at the breaker stage affects endogenous jasmonates (JAs), ethylene production, metabolic pathways, and flavor profiles in 'FL 47' tomatoes.

**Methods:**

Exogenous MeJA was applied to red 'FL 47' tomatoes at the breaker stage. We measured endogenous JAs (especially JA-Ile), ethylene production, enzyme activities (LOX, AOC, AOS, OPR, ACO, ACS), mRNA abundances of related genes (*SlLOXD*, *SlAOC*, *SlAOS*, *SlOPR3*, *SlACO1*, *SlACS2*, *SlACS4*, *SlMYC2*, *SlMED25*, *SlETR3*, *SlETR4*, *SlETR7*, *SlEIN2*, *SlEIL1*, *SlEBF1*, *SlEBF2*, *SlERF1*, *SlLOXC*, *SlPSY1*, *SlCCD1A*, *SlCCD1B*, *SlBCAT1*), production of sugars, organic acids, and 21 volatiles, as well as substrate contents (phytoene, phytofluene, trans-lycopene, γ-carotenoid, β-carotenoid, linoleic and linolenic acid) in MeJA-treated and control fruits.

**Results:**

MeJA treatment suppressed endogenous JAs (JA-Ile by 13%) and ethylene production by 33%. Enzyme activities in metabolic pathways were reduced to over 87% of control levels, and mRNA abundances of the aforementioned genes decreased by 17-30%. Production of sugars, organic acids, and volatiles was altered, leading to changes in flavor profile. Specifically, six key ethylene-regulated volatiles (geranyl acetone, 1-penten-3-one, 6-methyl-5-hepten-2-one, 2-methyl butanal, 3-methyl butanal, 3-methyl butanol) were reduced, concomitant with 19-29% lower mRNA abundances of biosynthetic genes (*SlLOXC*, *SlPSY1*, *SlCCD1A*, *SlCCD1B*, *SlBCAT1*) and substrate contents over 74% of control levels.

**Discussion:**

By considering the positive relationship between endogenous JA-Ile and ethylene levels in ripening fruit, our results imply that MeJA-mediated changes in aroma profile in red 'FL 47' tomatoes may result from mitigated endogenous JAs (especially JA-Ile) and ethylene biosynthesis and signaling transduction processes. These findings enhance understanding of hormone interactions in fruit quality formation and suggest potential applications for improving post-harvest tomato flavor.

## 1 Introduction

Tomato (*Solanum lycopersicum* L.) provides important nutrients in the human diet (Wang et al., 2023). Aroma quality determines the unique flavor of tomato and consumer acceptability (Klee and Giovannoni, 2011; Li et al., 2023; Wang et al., 2015). Over 400 volatile compounds have been identified in ripening tomatoes; however, only a small amount have been defined as important contributors to aroma quality (Li et al., 2023; Wang et al., 2015).

Tomato ripening is characterized by an increment of climacteric ethylene (Wang et al., 2016). Ethylene, a key endo-hormone synthesized from methionine via S-adenosylmethionine (AdoMet) synthetases, 1-aminocyclopropane-1-carboxylate synthase 2/4 (SlACS2/4), and oxidase 1 (SlACO1) (Jia et al., 2023). Upon synthesis, ethylene binds to several receptors, including ETHYLENE RESPONSE 3 (SlETR3), SlETR4, and SlETR7, inactivating constitutive triple response 1 (SlCTR1) and then promoting the cleavage of the carboxyl end in ethylene insensitive 2 (SlEIN2) (Liu et al., 2015; Wen et al., 2012). This stabilizes ethylene insensitive 3/ein3-like 1 (SlEIN3/SlEIL1) by inhibiting ein3-binding f-box protein 1/2 (SlEBF1/2). Finally, the accumulation of EIN3/EIL1 in nucleus activates downstream ethylene response factors (SlERFs) (Shan et al., 2022).

Climacteric ethylene plays a key role in aroma formation during ripening (Wang et al., 2016). Along with the increment of ethylene evolution during ‘FL 47’ fruit ripening, most key aroma contributors accumulated with a burst at later ripening stages (Jia et al., 2023; Klee, 2010; Wang et al., 2016). Antisense of *SlACS2* inhibited ethylene production and then the formation of acetone, methanol, ethanol, 1-penten-3-one, hexanal, *trans*-2-hexenal, *cis*-3-hexenal, 2 + 3-methylbutanol, *trans*-2-heptenal, 6-methyl-5-hepten-2-one, *cis*-3-hexenol, 2-isobutylthiazole, 1-nitro-2-phenylethane, and geranyl acetone in tomato (Baldwin et al., 2000). This phenomenon is likely due to the ethylene-mediated alteration of gene (e.g. *SlLOXC* for C5 & C6 volatiles) expression level, enzyme activities, and substrate (e.g. carotenoids for apocarotenoids) content in their biosynthesis pathways (Alba et al., 2005; Cao et al., 2024; Chen et al., 2004; Griffiths et al., 1999; Shen et al., 2014; Tieman et al., 2006; Wang et al., 2016).

Various postharvest handling techniques, such as low-temperature storage, methyl jasmonate (MeJA), and 1-methylcyclopropene (1-MCP) fumigation, etc., have been applied to extend shelf life of tomato fruit (Alonso-Salinas et al., 2024; Baldwin et al., 2011a; Deltsidis et al., 2015; Wang et al., 2016, 2015). Jasmonates (JAs), including jasmonic acid (JA), MeJA, and jasmonoyl-isoleucine (JA-Ile), regulate plant development and stress response (D'Onofrio et al., 2018; Zhu et al., 2022). Extensive studies have investigated the role of JAs in tomato ripening and then quality formation. Similar to salicylates (SAs) (Ding and Wang, 2003; Jia et al., 2023), the role of exogenous JAs treatment in fruit ripening is cultivar-, concentration- or ripening stage-dependent (Wang et al., 2021). For example, 0.50 mM MeJA soaking of mature green ‘Xin Taiyang’ tomato upregulated the expression of *SlACS2*/*4*, *SlACO1*, *SlETR3*/*4/6/7*, *SlEIN2*, *SlEIL2-4*, and *SlERF1*, and thus promoted ethylene production, therefore enhancing sucrose, total phenolics and flavonoids biosynthesis (Tao et al., 2021a, 2022, 2021). A similar result was observed in ‘ZheYingFen No1’ tomato after 0.05 μM MeJA soaking at mature green stage (Liu et al., 2018). On the other hand, preharvest 0.25 and 0.50 mM MeJA spray of mature green ‘Kumato’ tomato mitigated ethylene evolution, and thus maintained firmness and nutritional quality (Baek et al., 2023). Similar outcome was observed after 50 μM MeJA fumigation of breaker ‘FL 47’ tomato (Wang et al., 2015).

In higher plants, JAs formation is derived from α-linolenic acid (α-LeA) and hexadecatrienoic acid (HTA) by the sequential catalytic actions of lipoxygenase D (LoxD), allene oxide synthase (AOS), and allene oxide cyclase (AOC), producing *cis-*(+)- 12-oxophytodienoic acid (*cis*-(+)-OPDA) and dn-OPDA (Wu et al., 2011). After transportation into the peroxisome, *cis*-(+)-OPDA and dn-OPDA are catalyzed into (+)-7-iso-JA by several enzymes, such as 12-oxo-phytodienoic reductase 3 (OPR3), OPR2, OPC-8 coenzyme A ligase1 (OPCL1), etc. Finally, (+)-7-iso-JA, after epimerization into (-)-JA, is further converted into JA-Ile, the bioactive compound in JAs signaling transduction process (Ali and Baek, 2020).

After transportation into the nucleus via jasmonic acid transfer protein 1 (JAT1), JA-Ile binds to CORONATINE INSENSITIVE1 (COI1), an integral part of the Skp-Cullin-F-box (SCF) complex. Subsequently, COI1 targets JASMONATE-ZIM-DOMAIN proteins (JAZs) with the aid of inositol pentakisphosphate 5 (IP5) for poly-ubiquitination and subsequent degradation of JAZs by 26S proteome. Finally, MYC2 blockage was released to activate its downstream gene expression (Pauwels and Goossens, 2011; Wasternack and Hause, 2013). Furthermore, the initiation of transcription requires the interaction of MYC2 and MEDIATOR25 (MED25) subunit to recruit general transcription factors (GTFs) and RNA polymerase II (Ali and Baek, 2020; Gimenez-Ibanez et al., 2015). Recently, a small cluster of JA-inducible bHLH proteins, known as SlMYC2-targeted, bhlh 1 (SlMTB1), SlMTB2, and SlMTB3, were characterized from tomato, which play a negative role in JAs signaling transduction (Liu et al., 2019).

The above-mentioned findings facilitates the discovery of the proteins, which participate in the crosstalk between endogenous JAs (especially JA-Ile) and ethylene. Li et al. (2017) found that MdMYC2, whose transcription was induced by 100 μM MeJA spray, could interact with *MdACS1*, *MdACO1*, and *MdERF3* promoters and then activate their expression, thus promoting ethylene metabolism during ‘Orin’ apple ripening. Additionally, SlMED25 could form a transcriptional module with SlEIL1 to regulate the expression of the ripening-related regulatory as well as structural genes through promoter binding (Deng et al., 2023).

However, information on the mechanism of exogenous MeJA-mediated suppression of ethylene metabolism and thus quality formation in the ripening tomato fruit is still limited. By considering its role in JAs signaling transduction process, alteration of endogenous JA-Ile metabolism might be of great importance for us to understand the above-mentioned phenomenon (Fonseca et al., 2009; Zhu et al., 2024). In this study, ‘FL 47’ tomato at breaker stage was used as the material for H_2_O (control) and MeJA treatments. The alterations of endogenous JAs (especially JA-Ile), ethylene, and flavor metabolisms in the red fruit were detected. Moreover, the JAs-ethylene crosstalk as well as their function in key volatile production were determined as well.

## 2 Materials and methods

### 2.1 Plant materials

Uniform and defect-free ‘FL 47’ tomato fruits (a cultivar widely grown in Florida, USA), with an average weight of 248 g, were harvested at the mature green stage from a commercial orchard in Fort Pierce, Florida. The fruits were exposed to ethylene at 20°C to initiate and synchronize the ripening process. Fruits at the breaker stage were then selected based on USDA standards (USDA, 1991).

Subsequently, samples were divided into two groups for 50 μM MeJA and H_2_O (control) treatments. There were five biological replicates for each treatment. Each biological replicate included a batch of 30 fruits, which were treated independently according to the procedure of Wang et al. (2015) (**Supplementary Figure S1a**). Briefly, 30 fruits from each biological replicate were placed in a 45-L airtight glass container; then, filter paper disks (5 cm in diameter) soaked with MeJA or H_2_O were affixed to the top of the glass container and maintained at 20°C for 24 h (a final vapor concentration of 50 μM inside the container). To achieve the target headspace concentration of 50 μM, equivalent to 1.222 μL·L^-1^(ppm v/v), 0.505 g of pure MeJA was applied to a filter paper and allowed to evaporate fully within the container. MeJA treatment concentration and duration was selected based on the results of previous studies (Li et al., 2022; Min et al., 2020; Wang et al., 2015; Zhu et al., 2024). After fumigation, tomatoes were ripened at 20°C and 70-85% relative humidity (**Supplementary Figure S1a**). Fruits were sampled at the same time when the control and MeJA-treated fruits turned red based on the USDA standard (USDA, 1991) (7-d after treatment) (**Supplementary Figure S1a**). a^*^ value, firmness, and weight loss in the control and MeJA-treated fruits at red stage were 23.23 and 20.67, 11.12 N and 13.20 N, 5.32% and 5.09%, respectively (**Supplementary Figure S1b**).

For sampling, pericarp tissues from eight fruit per replicate were quickly removed with a sharp stainless-steel knife, immersed in liquid N_2_, fractured to pieces, and then stored at -80 °C for the analysis of metabolite, enzyme activities, gene expression profile, etc.; on the other hand, the remaining fruit were used for the determination of softening process, color development, ethylene evolution, flavor profile, etc.

### 2.2 Softening process and color development assay

The firmness assay was conducted using a food texture analyzer (Model 3200; Instron, Canton, MA) equipped with a round, flat-surfaced sensor measuring 9 cm in diameter (Jia et al., 2023).

The coloration of the fruits was quantified with a Minolta CR-400 chromameter (Osaka, Japan) at four equatorial locations (Jia et al., 2023). Prior to every measurement, a standard white tile was utilized to calibrate the device.

### 2.3 Ethylene evolution assessment

Ethylene evolution was assayed according to the method of Wang et al. (2019). Each replicate’s fruits were weighed before being incubated in a 2-L glass jar. After incubation at 20°C for 30 min, 5.0 mL of headspace atmosphere was extracted by a gas-tight syringe, and then analyzed by a gas chromatograph (HP 5890A, Hewlett Packard, Avondale, PA) fitted with a GSQ column. Gas constituents were identified and quantified by comparison of retention time and peak area with those in gas standard.

### 2.4 ACC analysis

ACC was analyzed based on the protocol of Lindo-García et al. (2020). Briefly, homogenization of 2.0 g pericarp sample was taken with 4 mL sulfosalicylic acid solution (5%, w/v). Following 30 min of agitation, centrifugation was applied to the blend for 10 min under a force of 8000 × g. Subsequently, 1.5 mL of the supernatant was collected and then incubated with 10 mmol·L^-1^ HgCl_2_, NaOCl and saturated NaOH (2:1, v/v). Finally, 1 mL of headspace gas was sampled and then injected into a gas chromatograph for ethylene assay.

### 2.5 JAs determination

Jasmonates (JA and JA-Ile) were determined by following the Zhu et al. (2024) method. Briefly, 1.0 g of pericarp tissue was homogenized in 5.0 mL of ice-cold extraction buffer (methanol: water: acetic acid, 80: 19: 1, v/v/v) and its filtration was taken through a double layer of Miracloth (Calbiochem, La Jolla, CA) and then spun down at 12,000 × g under 4°C for 20 min to obtain the supernatant. After drying with the aid of nitrogen, sample was redissolved in 400 μL methanol, and then filtered through a 0.22-μm Millipore filter (Siemens-Millipore, Shrewbury, MA) for JAs assay by a UPLC-MS/MS (Sciex Triple Quad 5500 LC-MS/MS, USA) system.

### 2.6 Sugar and organic acid analysis

Total soluble solids (TSS) were determined using a digital refractometer (ATAGO PR-101; Atago Co., Tokyo, Japan) (Li et al., 2021). For TA, sample was homogenized, filtered through two layers of Miracloth (Calbiochem, La Jolla, CA), and then centrifuged at 12,000 *g* for 20 min before collection of the supernatant for TA assay, using a titrator (808 Titrando; Metrohm, Riverview, FL, USA) (Li et al., 2021).

Individual sugars and organic acids were extracted and analyzed based on the method of Raithore et al. (2015), with some modifications. Briefly, 2.0 g of pericarp tissue was homogenized with ultrapure water, filtered through two layers of Miracloth (Calbiochem, La Jolla, CA), and then centrifuged at 12,000 *g* for 20 min at 4°C before collection of the supernatant to filtrate through a 0.45-μm Millipore filter (Siemens-Millipore, Shrewbury, MA). Individual sugar was analyzed by a high-performance liquid chromatography (HPLC) system, equipped with a Sugar-Pak column (10 μm, 300 mm × 6.5 mm; Waters, Milford, MA) and an Agilent 1100 series refractive index detector (Agilent Technologies, Santa Clara, CA). On the other hand, individual organic acid was assayed by a HPLC system with an AltechOA1000 Prevail organic acid column (9 μm, 300 mm × 6.5 mm; Grave Davison Discovery Sciences, Deerfield, IL) and a Spectra System UV 6000 LP photo diode array detector (Thermo Fisher Scientific, Waltham, MA). Identification and quantification of each component were conducted following the method of Raithore et al. (2015).

### 2.7 Headspace volatile analysis

Once a uniform mixture was obtained, 4.3 g pericarp tissue and 1.7 mL saturated CaCl_2_ were added to a vial supplied by Gerstel Inc. (Linthicum, MD). Next, volatile compounds were analyzed by using HS-SPME-GC-MS, based on the method by Jia et al. (2023). Sample was incubated for 30 min at 40 °C before exposure of a 2-cm solid phase microextraction (SPME) fiber (50/30 μm DVB/Carboxen/PDMS; Supelco, Bellefonte, PA) to the headspace for another 30 min at 40 °C. After exposure, the SPME fiber was inserted into the injector of a GC-MS (Model 6890, Agilent, Santa Clara, CA) to desorb the extract for 15 min at 250 °C. Data were collected using the ChemStation G1701 AA data system (Hewlett-Packard, Palo Alto, CA). Volatile compounds were identified quantified based on the method of Jia et al. (2023).

### 2.8 Fatty acid assay

Homogenization of the pericarp tissue occurred in a 2:1 (v/v) chloroform/methanol solution, after which it was filtered using two layers of Miracloth (Calbiochem, La Jolla, CA) and centrifuged at 5,000 × g for 5 min under 4°C conditions. The supernatant was then dried through a rotating vacuum evaporator.

Determination of fatty acids was performed using the fatty acid methyl ester (FAME) technique, as described by Ties and Barringer (2012). Briefly, esterification of the 0.5 g lipid sample, initially dissolved in chloroform, involved adding 10 mL of 4% methanolic-sulfuric acid and 1 mL of benzene, followed by a 2-h boil at 80-90°C; methyl esters were then extracted with the assistance of distilled water and hexane; finally, hexane layer was collected, and then evaporated under nitrogen before the addition of isooctane.

Fatty acid methyl esters (FAMEs) were evaluated via an Agilent 7890B gas chromatography system (Agilent Technologies Canada Inc.), incorporating a flame-ionization sensing unit (FID) alongside a capillary column of the Rtx-2330 type (30 m length, 0.32 mm ID, 0.20 μm thickness), composed of fused silica from Restek (Saini et al., 2017).

### 2.9 Carotenoid analysis

With some adjustments, the Alba et al. (2005) protocol was used to determine carotenoids. Briefly, 0.2 g pericarp tissue underwent extraction with magnesium carbonate and tetrahydrofuran/methanol. Afterwards, the homogenate was filtered through Spin-X centrifuge filters (0.45-mm nylon filter; Corning/Costar 8170), and tissue debris was reextracted with tetrahydrofuran to ensure complete extraction. The carotenoid/nonpolar phase was separated from the aqueous phase through two separation steps, first with petroleum ether and 25% NaCl and next with petroleum ether. Subsequently, the two upper phase aliquots were combined, dried down in a vacufuge (Eppendorf), and then passed through a syringe filter (GE Osmonics) before analysis.

Carotenoid determination was conducted via a Dionex HPLC apparatus, incorporating a PDA-100 photodiode array detector (Dionex, Idstein, Germany) and a YMC Carotenoid S-5 C30 column (4.6 × 250 mm; Waters) (Alba et al., 2005).

### 2.10 Free (iso)leucine determination

Free (iso)leucine were determined by the method of Jia et al. (2022) with some modifications. An automated system for amino acid analysis (Model L-8900, Hitachi, Tokyo, Japan) was used to evaluate 0.5 g of pericarp tissue that had been homogenized in a 4% (w/v) sulfosalicylic acid solution. Following collection, the supernatant underwent centrifugation at a speed of 12,000 × g for 20 min at 4°C. It was then sequentially filtered using two Miracloth layers (Calbiochem, La Jolla, CA) and a 0.22-μm Millipore filter (Siemens-Millipore, Shrewsbury, MA).

### 2.11 Electronic tongue determination

120 g of pericarp tissue, after homogenization and filtration through two layers of Miracloth (Calbiochem, La Jolla, CA). The homogenate was subjected to centrifugal separation at 12,000 g for 20 min while maintained at 4°C, followed by harvesting of the resulting supernatant. The e-tongue assay was conducted using the Astree II system for liquid analysis from Alpha MOS (Hanover, MD, USA). It features a reference electrode (Ag/AgCl), chemometric software, a 16 - position autosampler, and a sensor array (Raithore et al., 2015). The sensors of CA, JE, JB, HA, GA, BB and ZZ were all calibrated, and validated using Alpha MOS standards before testing.

### 2.12 Electronic nose assay

2.15 g of pericarp tissue, after homogenization with 0.85 mL of the saturated CaCl_2_, was transferred to a 10-mL vial. A FOX 4000 system (Alpha MOS, Toulouse, France) was then employed (Baldwin et al., 2012) for e-nose assay. Sample was incubated for 2 min at 40°C prior to headspace injection into the e-nose. E-nose data acquisition program was a 2-min sampling time followed by an 18-min delay between samples for sensor recovery.

### 2.13 Crude protein extraction and enzyme activity assay

#### 2.13.1 Ethylene-biosynthesis-related enzyme

A crude extract of ACS enzyme was obtained via homogenization of 10.0 g pericarp tissue with a buffer solution for extraction including Tricine at 200 mM (pH 8.5), DTT at 10 mM, pyridoxal phosphate at 20 μM, and polyvinylpyrrolidone (PVP) at 2% (w/v). Centrifugation of the homogeneous mixture was performed under a force of 18,000 × g for 20 min at 4°C. Subsequently, 2.5 mL of the supernatant was loaded into a Sephadex G-25 column (PD 10, Pharmacia, Madrid, Spain), which was previously equilibrated with 5 mM Tricine buffer (pH 8), 1 mM DTT, and 2 μM pyridoxal 5-phosphate. After elution with the same buffer, 1.5 mL of sample was incubated with 200 mM Tricine buffer (pH 8.0), 100 μM S-adenosyl-l-methionine (SAM) for 2 h at 25°C. The reaction was then stopped by addition of 100 mM HgCl_2_. Finally, 1 mL of sample was collected and mixed with 100 μL of NaOCl and saturated NaOH (2:1, v/v) prior to collection of headspace atmosphere. ACC activity was calculated by monitoring ethylene formation by a gas chromatograph (HP 5890A, Hewlett Packard, Avondale, PA) (Chiriboga et al., 2012).

10.0 g of tissue was homogenized for ACO activity using 20 mL of extraction buffer that contained glycerol at 10%, sodium ascorbate at 30 mM, DTT at 5 mM, PVP at 1% (w/v), plus 0.1 M Tris-HCl adjusted to pH 7.4. Following filtering, the homogenized sample underwent centrifugation at 16,000 × g at 4°C for 20 min. Subsequently, 2.5 mL from the supernatant was loaded into a Sephadex G-25 column, which was previously equilibrated with Tris-HCl at 20 mM (pH 7.4), 10% glycerol, sodium ascorbate at 3 mM, and DTT at 1 mM. After elution, 0.5 mL of the sample was blended with a solution containing 50 μM 1-aminocyclopropane-1-carboxylic acid (ACC), 3 mM sodium bicarbonate, and ferrous sulfate (FeSO_4_) at 10 μM, aired, and then incubated at 25°C for 20 min before collection of headspace atmosphere. ACO activity was calculated by monitoring ethylene formation by a gas chromatograph (HP 5890A, Hewlett Packard, Avondale, PA) (Chiriboga et al., 2012).

#### 2.13.2 JAs-biosynthesis-related enzyme

Lipoxygenase (LOX) activity was measured according to the method of Bai et al. (2011). 10 g of pericarp tissue was homogenized in an extraction solution of sorbitol at 250 mM, Tris-HCl at 150 mM (pH 8.0), MgCl_2_ at 10 mM, aminocaproic acid at 5 mM, DTT at 5 mM, glycerol at 1% (v/v), PVP at 0.2% (w/v), PMSF at 0.1 mM, and benzamidine at 0.1 mM. The homogenate was vortexed and then filtered through two layers of Miracloth (Calbiochem, La Jolla, CA). After that, the supernatant was taken after being centrifuged at 12,000 × g at 4°C for 20 min. LOX activity was determined by monitoring the formation of conjugated dienes at 234 nm, using an extinction coefficient of 25 mM^-^¹·cm^-^¹ (Bai et al., 2011).

AOS, AOC and OPR activities were measured based on the method of Zhu et al. (2024), with some modifications. Briefly, 0.5 g of pericarp tissue was homogenized using 5.0 mL of 50 mM PBS buffer (pH 7.4) and filtered through a double layer of Miracloth (Calbiochem, La Jolla, CA), and then centrifuged at 12,000 × g for 30 min under 4°C prior to collection of the supernatant. AOS, AOC, and OPR activities were quantified utilizing an ELISA assay kit designed for plants (Jiangsu Meimian Industrial Co., Ltd. in Jiangsu, China). One unit of enzyme activities was defined as the consumption of 1 μmol substrate in the absorbance of 450 nm per minute.

The crude extract’s protein content was calculated using Bradford (1976) technique.

### 2.14 Gene expression profile assay

The gene-specific primers were designed via Premier 6.0 software (**Supplementary Table S1**). Total RNA extraction, qRT-PCR, and first-strand cDNA synthesis were performed according to Wang et al. (2018) with minor modifications. Total RNA was isolated using TRizol Reagents (Invitrogen, USA) and treated with RNase-free DNase (Qiagen, USA). RNA purity and integrity were assessed prior to cDNA synthesis. First-strand cDNA was synthesized using TransScript^®^ One-Step gDNA Removal and cDNA Synthesis SuperMix (TRANSGEN, China).

Subsequently, qRT-PCR assay was completed with the aid of SYBR^®^ PrimeScript^™^ RT-PCR Kit (Perfect Real Time; Takara). *SlActin* and *SlGAPDH* were used as the housekeeping genes, and the relative gene expression was calculated based on the 2^-ΔΔCT^ method (Liu et al., 2020; Zhang et al., 2018).

### 2.15 Gene function validation in tomato fruit

#### 2.15.1 Transient overexpression of gene

Transient overexpression of genes in ‘MicroTom’ tomatoes was conducted based on the protocol of our previous study (Jia et al., 2023). The ORFs of *SlACO1*, *SlLOXD*, *SlMTB1*, and *SlMYC2* were amplified from ‘FL 47’ tomato (**Supplementary Table S1**), inserted into the pCAMBIA1300 vector, transformed into *Agrobacterium tumefaciens* strain GV3101, and then incubated at 28°C until OD_660_ ≈ 1.0. Afterwards, the bacterial strain were resuspended in infiltration buffer prior to injection into ‘MicroTom’ tomato through carpopodium tissue (Jia et al., 2023). Fruit infiltrated with empty pCAMBIA1300 vector were used as the control. There were three biological replicates per treatment, with ten fruit per biological replicate.

#### 2.15.2 Transient silence of gene

Transient silence of genes in ‘MicroTom’ tomatoes was conducted based on the method of our previous study (Jia et al., 2023). In brief, about 250-bp fragments of *SlACO1*, *SlLOXD*, *SlMTB1*, and *SlMYC2* ORFs were cloned from ‘FL 47’ tomato, and then introduced into pTRV2 vector (**Supplementary Table S1**). Afterwards, the constructed plasmid and pTRV1 were transformed into *A. tumefaciens* strain GV3101, respectively, resuspended in the infiltration buffer, and then slowly injected into ‘MicroTom’ tomato fruit through carpopodium tissue (Jia et al., 2023). Fruit infiltrated with empty pTRV2 and pTRV1 vectors were used as the control. There were three biological replicates per treatment, with ten fruit per biological replicate.

### 2.16 Statistical analysis

**Figure 1:**
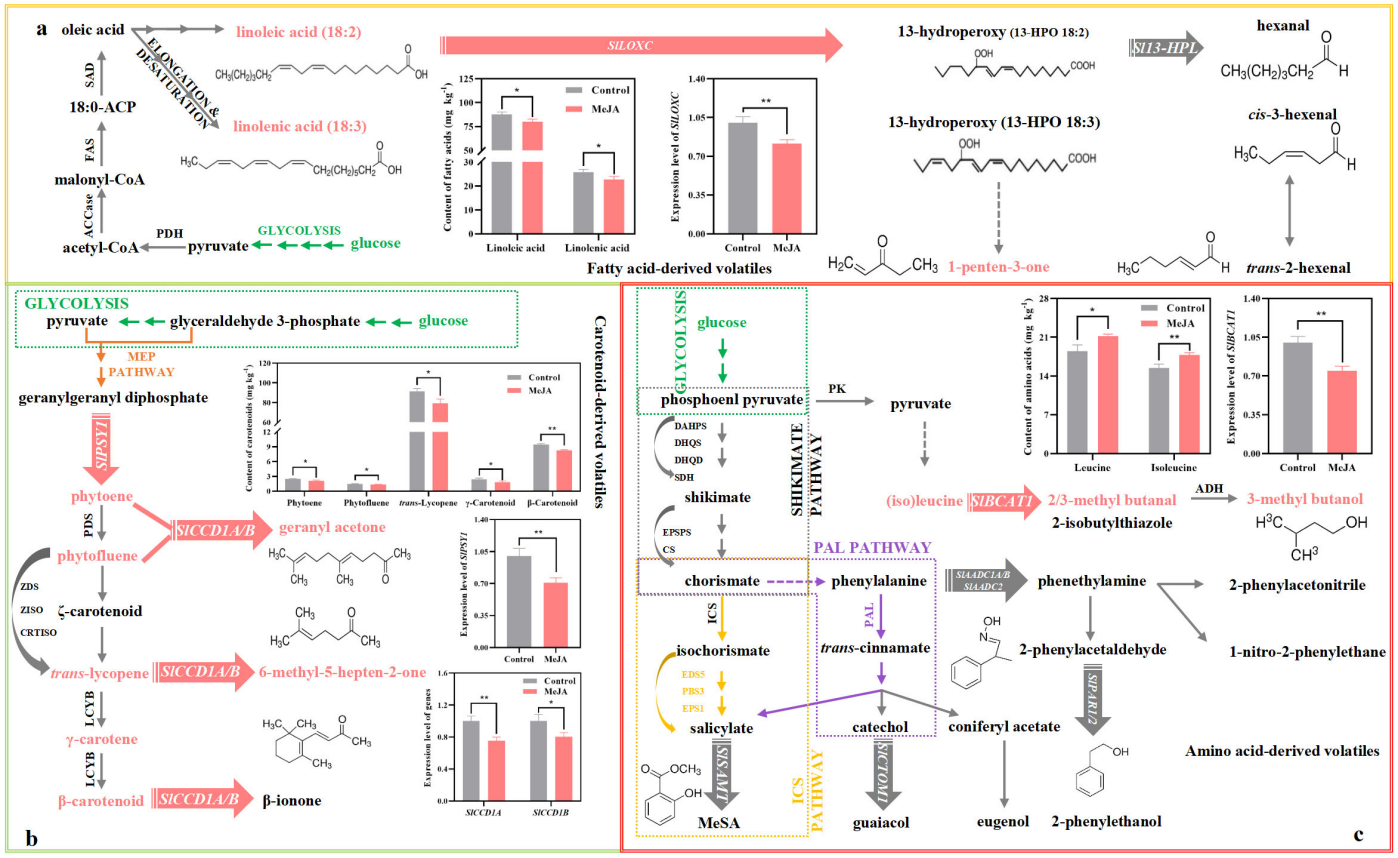
Impact of exogenous MeJA fumigation at breaker stage on the metabolism of six key volatiles in the red ‘FL 47’ tomato. **(a)** Fatty acid-derive volatile (1-penten-3-one). 1-penten-3-one is derived from linoleic and linolenic acids by the actions of *SlLOXC* (Shen et al., 2014). **(b)** Apocarotenoid volatiles (6-methyl-5-hepten-2-one and geranyl acetone). 6-methyl-5-hepten-2-one and geranyl acetone come from *trans*-lycopene and phytoene (or phytofluene), respectively, by the actions of *SlCCD1A/B* (Jia et al., 2023); and SlPSY1 is the key enzyme responsible for carotenoid biosynthesis (Wang et al., 2016). **(c)** Amino acid-derived volatiles (2-methyl butanal and 3-methyl butanal/ol). The formation of 2-methyl butanal and 3-methyl butanal/ol, which were derived from (iso)leucine, was initiated by *SlBCAT1* (Klee, 2010; Maloney et al., 2010). ‘FL 47’ tomato at breaker stage were treated with MeJA or H_2_O (control) for 24 h prior to ripening at 20°C; fruit were sampled at the same time when the control and MeJA-treated fruits turned red. The expression level of each gene in the control fruit was set as 1.0 based on qRT-PCR result. Data represent means ± standard deviation (SD) from three biological replicates; and significant differences (*p < 0.05; **p < 0.01) were determined via one-way ANOVA followed by Tukey’s HSD *post-hoc* test. The metabolic pathways of six key volatiles were drawn based on previous reports (Chen et al., 2009; Jia et al., 2023; Kachanovsky et al., 2012; Klee, 2010; Kössler et al., 2021; Maloney et al., 2010; Rambla et al., 2013; Wang et al., 2016). Abbreviations: ACCase, acetyl-CoA carboxylase; ACP, acyl carrier protein; ADH, alcohol dehydrogenase; CRTISO, carotenoid *cis-trans* isomerase; CS,chorismate synthase; DAHPS, 3-deoxy-d-arabino-heptulosonate-7-phosphate synthase; DHQD, 3-dehydroquinate dehydratase; DHQS, 3-dehydroquinate synthase; EDS5, enhanced disease susceptibility 5; EPS1, enhanced pseudomonas susceptibility 1; EPSPS, 5-enolpyruvylshikimate-3-phosphate synthase; FAS, fatty acid synthase; ICS, isochorismate synthase; LCYB, lycopene β-cyclase; PAL, phenylalanine ammonia-lyase; PBS3, avrPphB susceptible 3; PDH, pyruvate dehydrogenase; PDS, phytoene desaturase; PK, pyruvate kinase; SAD, stearoyl-acyl carrier protein Δ 9 desaturase; SDH, shikimate-5-dehydrogenase; ZDS, ζ-carotene desaturase; ZISO, ζ-carotene isomerase.

Statistical analyses were performed using one-way ANOVA followed by Tukey’s Honest Significant Difference (HSD) test to determine significant differences among treatments. Data represented the mean of three biological replicates, except for e-tongue/e-nose assay (five biological replicates), and significance levels were denoted as *p* < 0.05, *p* < 0.01. Error bar represented the standard deviation (SD) of the mean of three replicates. For volatile endpoints, *p*-values obtained from ANOVA/Tukey tests were adjusted using the Benjamini-Hochberg false discovery rate (FDR) procedure (*q* < 0.05). For the six key volatiles highlighted in **Figure 1**, treatment effects were further summarized using Cohen’s d with 95% confidence intervals estimated by bootstrap resampling (10,000-20,000 draws) (**Supplementary Table S3**). These adjustments were applied to enhance the robustness of the statistical interpretation while retaining the original ANOVA/Tukey analytical framework.

For e-nose and e-tongue analyses, AlphaSOFT (Alpha MOS) software was used to perform principal component analysis (PCA) and calculate the pattern discrimination index, expressed as the Mahalanobis distance (MD). The MD quantifies the multivariate separation between treatment groups by comparing the distance between group centroids relative to within-group variance. Higher MD values indicate stronger compositional or sensory matrix differences, whereas near-zero MD values reflect highly similar profiles (Baldwin et al., 2011b; Raithore et al., 2015).

## 3 Results

### 3.1 Impact of exogenous MeJA fumigation on JAs metabolism

**Figure 2:**
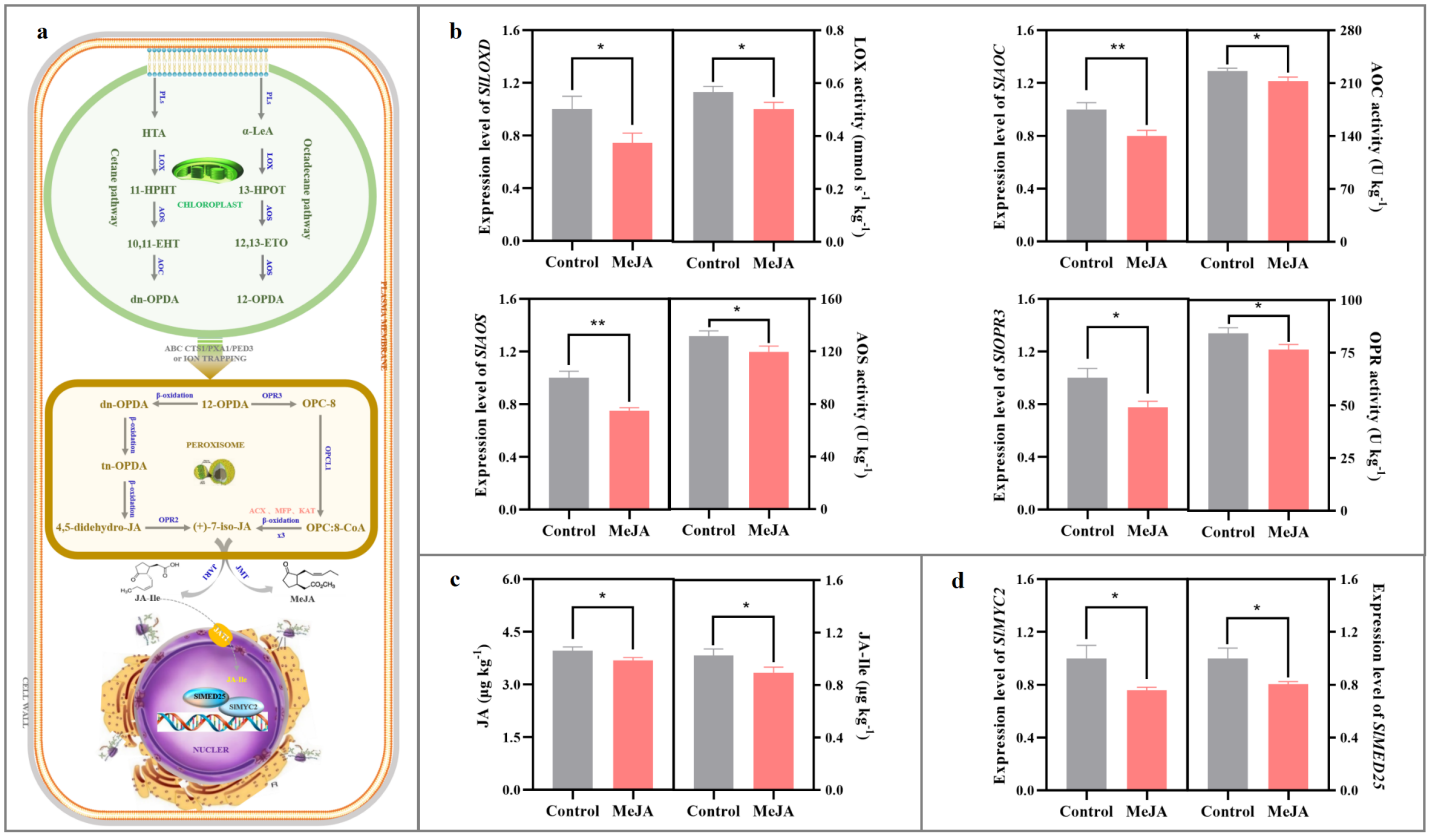
Impact of exogenous MeJA fumigation at breaker stage on JAs metabolism in the red ‘FL 47’ tomato. **(a)** JAs biosynthesis & signaling transduction pathways. In higher plant, JAs formation is derived from α-linolenic acid (α-LeA) and hexadecatrienoic acid (HTA) by the sequential catalytic actions of lipoxygenase D (LoxD), allene oxide synthase (AOS), and allene oxide cyclase (AOC), producing *cis-*(+)-12-oxophytodienoic acid (*cis*-(+)-OPDA) and dn-OPDA (Wu et al., 2011). After transportation into the peroxisome, *cis*-(+)-OPDA and dn-OPDA are catalyzed by several enzymes, such as 12-oxo-phytodienoic reductase 3 (OPR3), OPR2, OPC-8 coenzyme A ligase1 (OPCL1), etc., into (+)-7-iso-JA, which after epimerization into (-)-JA is further converted into various derivatives (Ali and Baek, 2020). JA-Ile has been characterized as the bioactive compound involved in JAs signaling transduction process (Fonseca et al., 2009). After transportation into the nucleus via jasmonic acid transfer protein 1 (JAT1), JA-Ile could bind to CORONATINE INSENSITIVE1 (COI1), an integral part of the Skp-Cullin-F-box (SCF) complex. Subsequently, COI1 targets JASMONATE-ZIM-DOMAIN proteins (JAZs) with the aid of inositol pentakisphosphate 5 (IP5) for poly-ubiquitination and subsequent degradation of JAZs by 26S proteome. Finally, MYC2 blockage was released to activate its downstream gene expression, such as *LoxD* and *threonine deaminase* (*TD*), etc (Pauwels and Goossens, 2011; Wasternack and Hause, 2013). Additionally, the initiation of transcription requires the interaction of MYC2 and MEDIATOR25 (MED25) subunit in the Mediator complex to recruit general transcription factors (GTFs) and RNA polymerase II (Ali and Baek, 2020; Gimenez-Ibanez et al., 2015). **(b)** Gene (*SlLOXD*, *SlAOC*, *SlAOS*, and *SlOPR3*) expression profiles and enzyme (LOX, AOC, AOS, and OPR) actives in JAs biosynthesis pathway. **(c)** Endogenous JA and JA-Ile contents. **(d)** Gene (*SlMYC2* and *SlMED25*) expression profiles in JAs signaling transduction pathway. ‘FL 47’ tomato at breaker stage were treated with MeJA or H_2_O (control) for 24 h prior to ripening at 20 °C; fruits were sampled at the same time when the control and MeJA-treated fruits turned red. The expression level of each gene in the control fruit was set as 1.0 based on qRT-PCR result. Data represent means ± standard deviation (SD) from three biological replicates; and significant differences (*p < 0.05; **p < 0.01) were determined via one-way ANOVA followed by Tukey’s HSD *post-hoc* test. Abbreviations: ACX, acyl-CoA oxidase; AOC, allene oxide cyclase; AOS, allene oxide synthase; 4,5-didehydro-JA, 4,5-didehydro-jasmonic acid; dn-OPDA, dinor OPDA; 10,11-EHT, 10,11(S)-epoxy-hexadeca (tri) enoic acid; 12,13-EOT, 12,13-(S)- epoxy-octadecatrienoic acid; 11-HPHT, 11-hydro- peroxyhexadecatrie- noic acid; 13-HPOT, 13-hydroperoxyoctadecatrienoic acid; HTA, hexadecatrienoic acid; (+)-7-iso-JA, (+)-7-iso-jasmonic acid; JA-Ile, jasmonoyl-isoleucine; JAR1, jasomonate resistant 1; JMT, jasmonic acid carboxyl methyltransferase; KAT, 3-ketoacyl-CoA thiolase; α-LeA, α-linolenic acid; LOX, lipoxygenases; OPC-8, 3-oxo-2-((Z)-pent-2-en-1-yl) cyclopentane-1- octanoic acid; MeJA, methyl jasmonate; MFP, multifunctional protein; OPC, 8-CoA: 3-oxo-2-((Z)-pent-2-en-1-yl)cyclopentane-1-octanoyl-CoA; OPCL1, OPC-8 coenzyme a ligase1; 12-OPDA, 12-oxo-phytodienoic acid; OPR, OPDA reductase; PLs, phospholipases; tn-OPDA, tetranor-OPDA.

As shown in **Figures 2a, b**, exogenous MeJA fumigation at the breaker stage downregulated *SlLOXD*, *SlAOC*, *SlAOS*, and *SlOPR3* mRNA abundances in the red ‘FL 47’ tomato by 20-26%; meanwhile, LOX, AOS, AOC, and OPR activities in the MeJA-treated tomato were more than 89% of those in the control fruit. The aforementioned phenomenon resulted in lower levels of endogenous JA and JA-Ile compared to those in the control fruit (**Figure 2c**).

Meanwhile, the expression levels of *SlMYC2* and *SlMED25* in the JA-Ile signaling pathway were also inhibited in the MeJA-treated fruit (**Figure 2d**).

### 3.2 Impact of exogenous MeJA fumigation on ethylene metabolism

**Figure 3:**
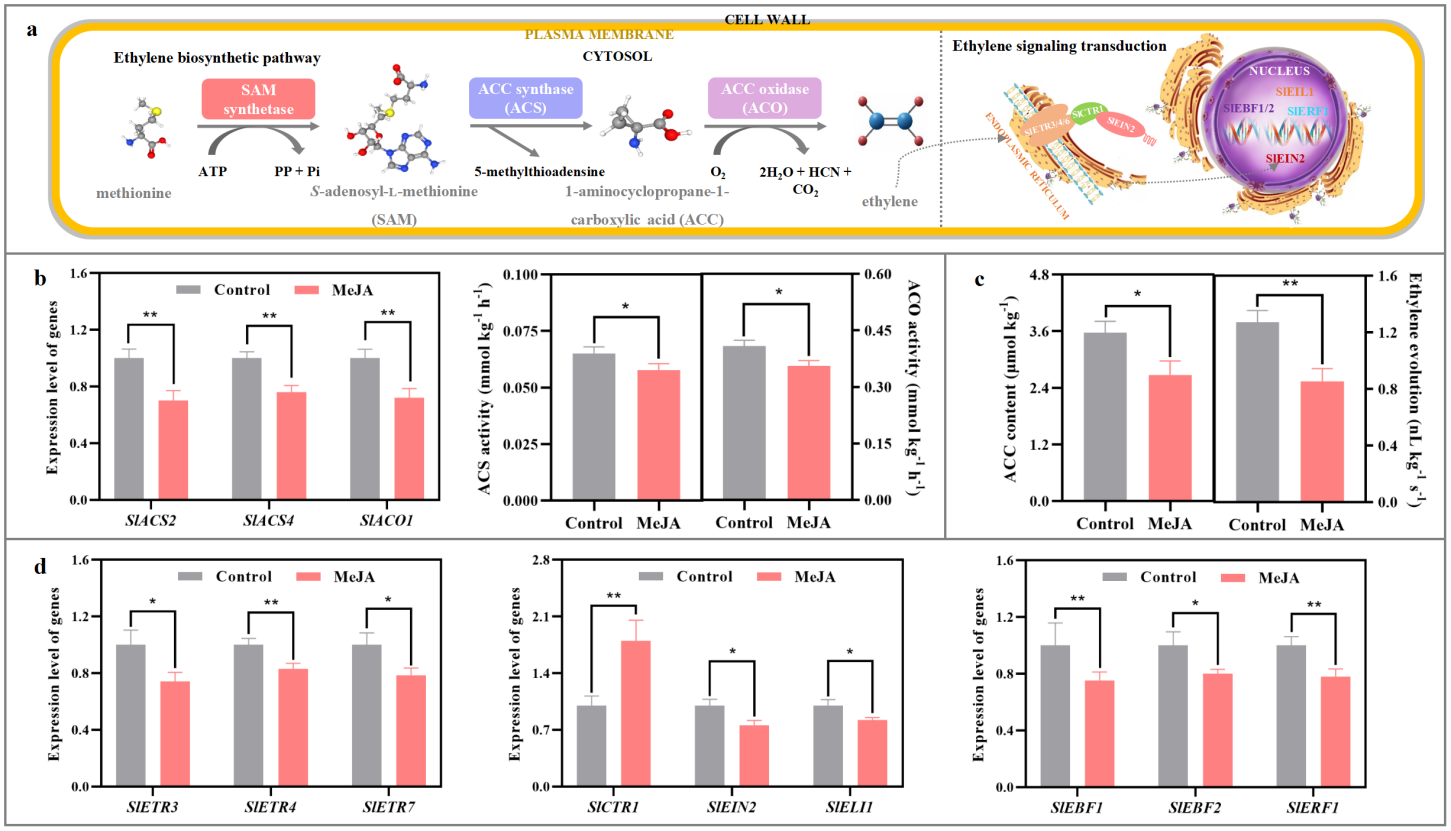
Impact of exogenous MeJA fumigation at breaker stage on ethylene metabolism in the red ‘FL 47’ tomato. **(a)** Ethylene biosynthesis & signaling transduction pathways. Climacteric ethylene, a key endo-hormone involved in tomato ripening process, is synthesized from methionine by the actions of S-adenosylmethionine (AdoMet) synthetases, 1-aminocyclopropane-1-carboxylic acid synthase 2/4 (SlACS2/4), and 1-aminocyclopropane-1-carboxylic acid oxidase 1 (SlACO1) (Jia et al., 2023). After formation, it could bind to several ethylene receptors, including SlETR3, SlETR4, and SlETR7, with the aid of cuprous ion (Cu^+^) (Liu et al., 2015). Such interaction causes the inactivation of constitutive triple response 1 (SlCTR1), which then promotes the cleavage of the carboxyl end in ethylene in-sensitive 2 (SlEIN2) (Wen et al., 2012). Then, the cleaved EIN2 enters the nucleus, and inhibits the ubiquitination and degradation of ethylene insensitive 3/ein3-like 1 (SlEIN3/SlEIL1), which is regulated by EIN3-binding F-box protein 1/2 (SlEBF1/2). Finally, the accumulation of EIN3/EIL1 in nucleus promotes the expression of downstream ethylene response factors (SlERFs) (Shan et al., 2022). **(b)** Gene (*SlACO1*, *SlACS2*, and *SlACS4*) expression profiles and enzyme (ACS, ACO) actives in ethylene biosynthesis pathway. **(c)** ACC and ethylene abundances. **(d)** Gene (*SlETR3*, *SlETR4*, *SlETR7*, *SlCTR1*, *SlEIN2*, *SlEIL1*, *SlEBF1*, *SlEBF2* and *SlERF1*) expression profiles in ethylene signaling transduction pathway. ‘FL 47’ tomato at breaker stage were treated with MeJA or H_2_O (control) for 24 h prior to ripening at 20°C; fruit were sampled at the same time when the control and MeJA-treated fruits turned red. The expression level of each gene in the control fruit was set as 1.0 based on qRT-PCR result. Data represent means ± standard deviation (SD) from three biological replicates; and significant differences (*p < 0.05; **p < 0.01) were determined via one-way ANOVA followed by Tukey’s HSD *post-hoc* test.

As shown in **Figures 3b, c**, when compared with those in the control fruit, the expression levels of *SlACS2*, *SlACS4*, and *SlACO1* in the MeJA-treated tomato were reduced by 24-30%; ACS and ACO activities suffered 12-13% reduction by MeJA fumigation; furthermore, ACC content in the treated fruit decreased by 25%.

In addition, gene expression profile in ethylene signaling transduction was also altered by MeJA treatment. As illustrated in **Figure 3d**, the mRNA abundances of *SlETR3*, *SlETR4*, *SlETR7*, *SlCTR1*, *SlEIN2*, *SlEIL1*, *SlEBF1*, *SlEBF2* and *SlERF1* in the MeJA-treated fruit were over 74% of those in the control fruit at the red stage.

### 3.3 Impact of exogenous MeJA fumigation on flavor profile

#### 3.3.1 Taste profile

**Figure 4:**
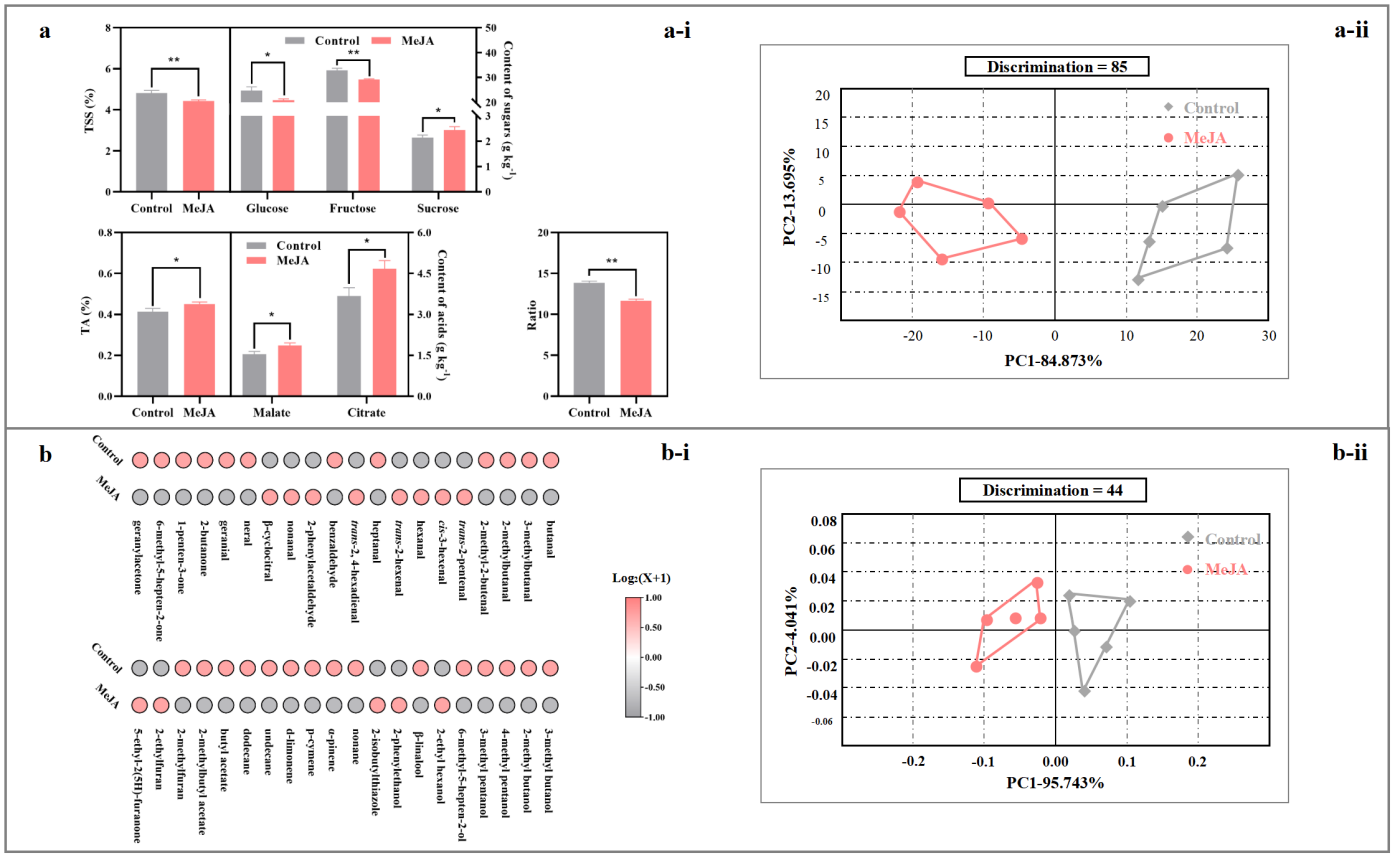
Impact of exogenous MeJA fumigation at breaker stage on flavor profile in the red ‘FL 47’ tomato. **(a)** Taste profile: **(a-i)** abundances of taste contributors, including sugars (glucose, fructose, and sucrose) and organic acids (malate and citrate); **(a-ii)** e-tongue result. **(b)** Aroma profile: **(b-i)** abundances of volatiles of 40 volatile components; **(b-ii)** e-nose result. ‘FL 47’ tomato at breaker stage were treated with MeJA or H_2_O (control) for 24 h prior to ripening at 20 °C; fruit were sampled at the same time when the control and MeJA-treated fruits turned red. Data represent means ± standard deviation (SD) from three biological replicates, except for e-nose or e-tongue assay (five biological replicates); and significant differences (*p < 0.05; **p < 0.01) were determined via one-way ANOVA followed by Tukey’s HSD *post-hoc* test. Color scale represents normalized log_2_-transformed (mean value of volatile abundance + 1), where red indicates a high level, grey indicates a low level, and white indicates a medium level. For e-nose and e-tongue data assay, the manufacturer’s statistical program, AlphaSOFT (Alpha MOS), was used; moreover, PCA, discrimination power of the auto-selected sensors, distance and pattern discrimination index between samples were also determined (Baldwin et al., 2011b; Raithore et al., 2015).

TSS and TA demonstrated opposite alteration after exogenous MeJA fumigation, causing the decrease in the ratio of TSS/TA (**Figure 4a-i**). For individual taste contributors, the abundances of glucose and fructose in the red tomato suffered 15% and 11%, respectively, reduction by MeJA fumigation; on the other hand, sucrose, malate, and citrate contents in the treated tomato were 14-27% higher than those in the control fruit (**Figure 4a-i**).

The electronic tongue (e-tongue) represents an alternative objective method for detecting variations in taste profiles (Raithore et al., 2015). The raw data obtained from the e-tongue analysis were multidimensional (7D) and thus subjected to PCA. As shown in **Figure 4a-ii**, the control fruit was clearly separated from MeJA-treated fruit along PC1, which explained 84.87% of total variance, with a Mahalanobis distance (MD) = 36.18, indicating a strong overall difference in liquid-phase taste matrix between treatments. A large Mahalanobis distance indicates strong matrix differences in liquids (e-tongue).

#### 3.3.2 Aroma profile

A total of 40 aromatic volatiles were identified from the red ‘FL 47’ tomato, including 16 aldehydes, 8 alcohols, 6 hydrocarbons, 4 ketones, 3 oxygen-containing heterocyclic compounds, 2 esters, and 1 sulfur- and nitrogen- containing heterocyclic compound (**Figure 4b-i**, **Supplementary Table S2**). **Supplementary Table S2** summarized their retention indexes (RIs), odor descriptions (Acree and Arn, 2010; Klee, 2010), odor thresholds in water (Van Gemert, 2003), and concentrations in the red fruit.

As shown in **Supplementary Table S2**, ketones suffered a 36% reduction after exogenous MeJA fumigation. Consistently, butanal, 3-methyl butanal, 2-methyl butanal, 2-methyl-2-butenal, heptanal, neral, geranial, 3-methyl butanol, 2-methyl butanol, 4-methyl pentanol, 3-methyl pentanol, 6-methyl-5-hepten-2-ol, undecane, dodecane, 2-butanone, 1-penten-3-one, 6-methyl-5-hepten-2-one, geranyl acetone, 2-methylbutyl acetate, and 2-methyl furan were lower in the MeJA-treated fruit than those in the control, while *trans*-2, *trans*-4-hexadienal displayed a opposite change (**Figure 1b-i**, **Supplementary Table S2**).

E-nose analysis could crudely mimic the mammalian olfactory system (Baldwin et al., 2011b). In this study, five sensors (LY2/LG, LY2/G, LY2/AA, LY2/GH, and LY2/gCTl) were selected through the AlphaSOFT sensor optimization module. PCA based on covariance was then performed. As shown in **Figure 4b-ii**, the first two principal components explained 99.78% of total variance, and the MD = 0.11, signifying only a subtle but detectable separation of headspace volatiles (McLachlan, 2005). Despite the small MD, samples were consistently separated along PC1 (positive vs. negative direction), suggesting minor yet reproducible alterations in volatile patterning consistent with GC-MS evidence (**Figure 4b-ii**).

### 3.4 Impact of exogenous MeJA fumigation on key volatile metabolism

Six out of 21 volatiles, whose formation was impacted by exogenous MeJA fumigation, were classified as key aroma contributors (**Figure 1**, **Supplementary Table S2**). They were derived from different metabolic pathways (Klee, 2010). After FDR correction, all six driver volatiles (**Figure 1**) remained significantly different between treatments. Standardized effect sizes (Cohen’s d) were large across compounds (≈ 3.06-5.48), with bootstrap 95% CIs that excluded zero despite the small sample size (**Supplementary Table S3**). These results indicate strong, directionally consistent shifts for the highlighted aroma-active volatiles under MeJA treatment.

#### 3.4.1 Fatty acid-derived volatile

1-Penten-3-one, known for its ‘fruity’, ‘floral’, or ‘green’ aroma (**Supplementary Table S2**) (Acree and Arn, 2010; Klee, 2010), is derived from linoleic and linolenic acids by the actions of *SlLOXC* (**Figure 1a**) (Shen et al., 2014). In this study, exogenous MeJA fumigation suppressed the mRNA abundance of *SlLOXC* by 19% (**Figure 1a**); meanwhile, the contents of linolenic and linoleic acids in the treated fruit suffered 9-12% reduction by MeJA treatment (**Figure 1a**).

#### 3.4.2 Apocarotenoid volatile

Geranyl acetone and 6-Methyl-5-hepten-2-one are categorized as ‘floral’, ‘citrusy’, ‘sweet’, or ‘estery’ (**Supplementary Table S2**) (Acree and Arn, 2010). They come from *trans*-lycopene and phytoene (or phytofluene), respectively, by the actions of carotenoid cleavage dioxygenases 1A (SlCCD1A) and SlCCD1B (**Figure 1b**) (Jia et al., 2023); and phytoene synthase 1 (SlPSY1) is the key enzyme governing the carotenoid biosynthesis (**Figure 1b**) (Wang et al., 2016). As shown in **Figure 1b**, exogenous MeJA fumigation inhibited the production of phytoene, phytofluene, *trans*-lycopene, γ-carotenoid, and β-carotenoid by 11-26%; moreover, the mRNA abundances of *SlPSY1*, *SlCCD1A*, and *SlCCD1B* in the MeJA-treated fruit were only 71-80% of those in control fruit (**Figure 1b**).

#### 3.4.3 Branched-chain volatile

2-Methyl butanal and 3-methyl butanal/ol, which were derived from (iso)leucine, impart ‘malt’, ‘cocoa’, ‘almond’, ‘whiskey’, or ‘burnt’ note to fruit (**Supplementary Table S2**) (Acree and Arn, 2010). Their formation was initiated by branched-chain aminotransferase 1 (SlBCAT1) (**Figure 1c**) (Klee, 2010; Maloney et al., 2010). As shown in **Figure 1c**, the production of leucine and isoleucine was enhanced by 15% after MeJA treatment; however, the expression level of *SlBCAT1* in the treated fruit was downregulated by 25% (**Figure 1c**).

### 3.5 Relationship between endogenous JA-Ile and ethylene levels in the ripening fruit

Then, we attempted to assayed the relationship between endogenous JA-Ile and ethylene.

**Figure 5:**
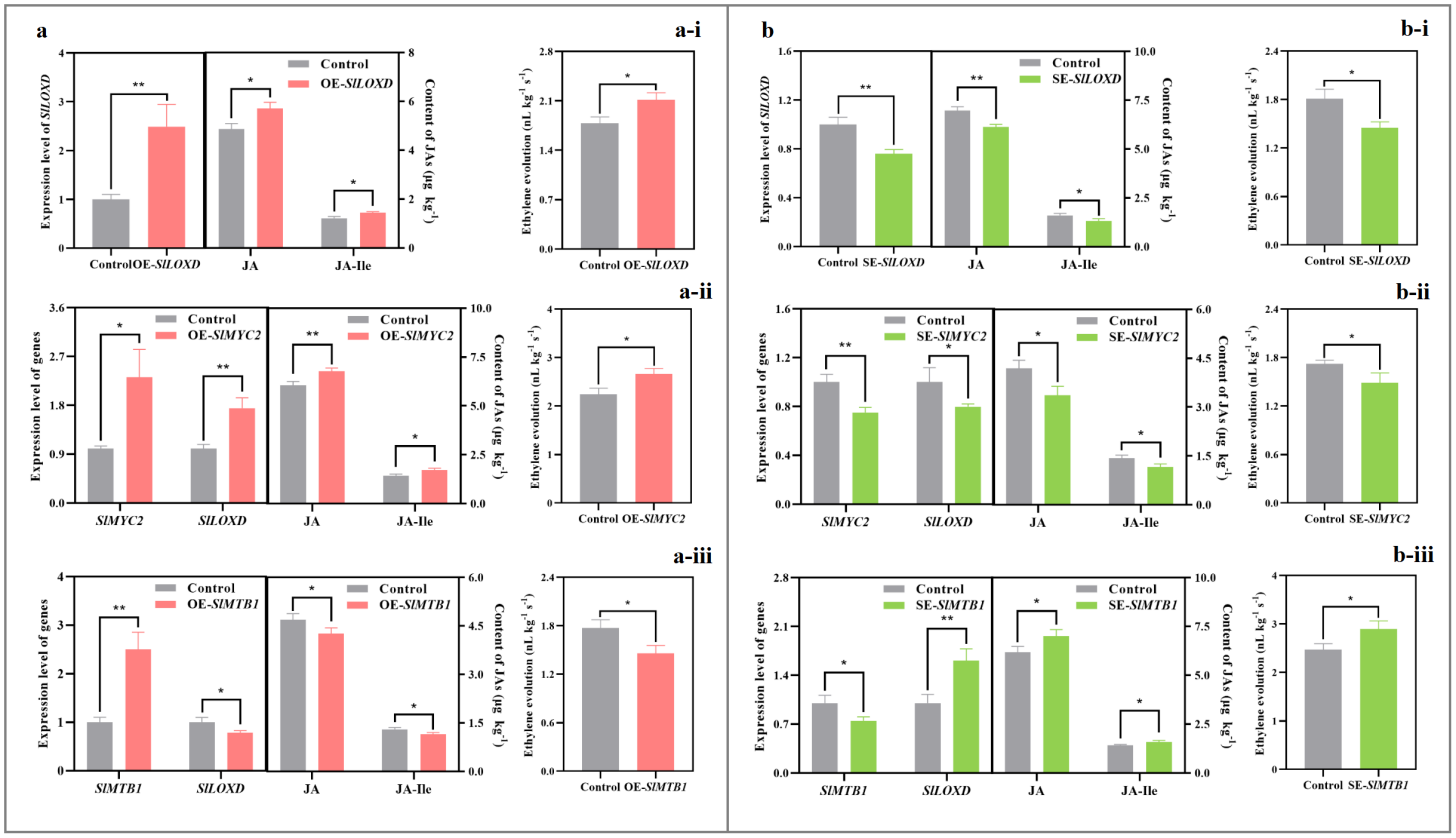
Impact of transient transformation of tomato with the JAs-metabolism-related genes on ethylene evolution. **(a)** Transient overexpression of *SlLOXD***(a-i)**, *SlMYC2***(a-ii)**, and *SlMTB1 ***(a-iii)** genes. *SlLOXD*, *SlMYC2*, and *SlMTB1* ORFs without stop codons were amplified and then inserted into the pCAMBIA1300 vector; afterwards, the recombinant vector was then transformed into *A*. *tumefaciens* strain GV3101 before injection into ‘MicroTom’ tomato through the carpopodium tissue. Fruit infiltrated with empty pCAMBIA1300 vector were used as the control. **(b)** Transient silence of *SlLOXD***(b-i)**, *SlMYC2***(b-ii)**, and *SlMTB1 ***(b-iii)** genes. About 250-bp fragments of *SlLOXD*, *SlMYC2*, and *SlMTB1* ORFs were amplified from ‘FL 47’ tomato and then inserted into pTRV2 vector; afterwards, the constructed plasmid and pTRV1 were transformed into *A. tumefaciens* strain GV3101, respectively, and then combined in a ratio of 1:1 before injection into ‘MicroTom’ tomato fruit through the carpopodium tissue. Fruit infiltrated with empty pTRV2 and pTRV1 vectors were used as the control. Pericarp tissue was collected after 3-d preservation at 20°C. The expression level of each gene in the control fruit was set as 1.0 based on qRT-PCR result. Data represent means ± standard deviation (SD) from three biological replicates; and significant differences (*p < 0.05; **p < 0.01) were determined via one-way ANOVA followed by Tukey’s HSD *post-hoc* test.

Transient overexpression of *SlLOXD* and *SlMYC2* genes (**Figures 5a-i, a-ii**) or transient silence of *SlMTB1* gene (**Figure 5b-iii**) upregulated *SlLOXD* transcription, promoted endogenous JA and JA-Ile accumulation, and thus elevated ethylene evolution in the ripening tomato. Conversely, an opposite result was detected in the *SlLOXD*/*SlMYC2*-silenced (**Figure 5b-i, 5b-ii**) or *SlMTB1*-overexpressing fruit (**Figure 5a-iii**).

Moreover, overexpression of *SlMYC2* also enhanced the production of six key volatiles in tomato. As shown in **Supplementary Figure S2a** and **Supplementary Table S4**, the abundances of 1-penten-3-one, geranyl acetone, 6-methyl-5-hepten-2-one, 2-methyl butanal, 3-methyl butanal, 3-methyl butanol in the control and *SlMYC2*-overexpressing fruit were 0.038 and 0.055, 0.25 and 0.40, 0.72 and 1.28, 1.03 and 1.32, 0.80 and 1.05, 0.24 and 0.36 mg·kg^-1^, respectively. On the other hand, an opposite phenomenon was observed in the silenced fruit (**Supplementary Figure S2b**, **Supplementary Table S4**).

### 3.6 Role of ethylene in key volatile metabolism

Then, we validated the involvement of ethylene in the generation of the above-mentioned six key volatiles.

As shown in **Supplementary Figures S3a, b**, transient overexpression of *SlACO1* gene considerably elevated ethylene evolution in tomato. Meanwhile, the production of phytoene, phytofluene, *trans*-lycopene, γ-carotenoid, β-carotenoid, linolenic and linoleic acids as well as the transcription of *SlLOXC*, *SlPSY1*, *SlCCD1A*, *SlCCD1B*, and *SlBCAT1* genes were upregulated in the overexpressing fruit (**Supplementary Figure S3c-e**). These outcome caused higher abundances of six key volatiles than those in the control (**Supplementary Figures S3c-e**).

An opposite phenomenon was detected after silence of *SlACO1* gene in fruit, which would suppress ethylene evolution (**Supplementary Figures S4a, b**). When compared with those in the control, substrate (carotenoids and fatty acids) contents, gene expression levels, and volatile production were downregulated in the *SlACO1*-silenced fruit (**Supplementary Figures S4c–e**; **Supplementary Table S5**).

## 4 Discussion

MeJA have been applied in the postharvest handling practice of tomato (Li et al., 2022). In agreement with the results from previous studies (Baek et al., 2023; Wang et al., 2015), exogenous application of 50 μM MeJA at the breaker stage delayed the ripening of process of ‘FL 47’ fruit, as demonstrated by the suppressed color development and softening process (**Supplementary Figure S1**). Moreover, the flavor profiles were altered as well. The accumulation of TSS, glucose, fructose, and 20 aromatic volatiles was inhibited by MeJA fumigation, which promoted sucrose, TA, malate, and *trans*-2, *trans*-4-hexadienal in the red fruit (**Figure 4**, **Supplementary Table S2**). Similar phenomenon was observed in a previous study (Wang et al., 2015).

Although sugars and organic acids play a crucial role in taste quality, the unique flavor of tomato depends on the complex mixture of aromatic volatiles (Klee and Giovannoni, 2011). Volatile concentration would substantially affect tomato liking independent of sugars or organic acids (Frick et al., 2023). Of > 400 volatiles in the ripening fruit, only nineteen have present in sufficient quantities to impact tomato aroma quality (Klee, 2010). Of 21 volatiles impacted by MeJA treatment, six were characterized as key aroma contributors (**Figures 4b-i**; **Supplementary Table S2**) (Klee, 2010). Further study uncovered that gene (*SlLOXC*, *SlBCAT1*, *SlPSY1*, and *SlCCD1A*/*B*) transcription and substrate (carotenoids and fatty acids) production in their biosynthesis pathways were mitigated, implying that these factors might be responsible for their reduction (**Figure 1**). In agreement with this, overexpression of *SlCCD1A/B* promoted apocarotenoid volatile formation in tomato (Cheng et al., 2021; Jia et al., 2023), while an opposite phenomenon was observed after mutation of *SlPSY1* (Cao et al., 2024). Similarly, the production of branched-chain volatiles, including 2-methyl butanol and 3-methyl butanol, increased in the *SlBCAT1-*overexpressing fruit (Kochevenko et al., 2012).

One limitation of this study is the sampling strategy, where fruits were sampled at the same time when the control and MeJA-treated fruits turned red (USDA, 1991), resulting in control fruits being redder at the same clock time (a* ≈ 23.23 vs 20.67) (**Supplementary Figure S1**). This maturity offset, while defensible for capturing treatment effects at a comparable ripening stage for MeJA-treated samples, may introduce biases in volatiles, sugars/acids, and gene expression due to differences in physiological maturity. To address this, no additional analyses were repeated after matching samples by an objective color threshold (e.g., a*). Therefore, future studies could incorporate such color-matched sampling to further validate the abovementioned findings.

Ripening of tomato fruit is a complex and highly coordinated developmental process, where the climacteric ethylene played a key role (Alexander and Grierson, 2002; Wang et al., 2016). In agreement with the finding in ‘Kumato’ tomato (Baek et al., 2023), exogenous MeJA fumigation of breaker ‘FL 47’ tomato inhibited *SlACO1* and *SlACS2/4* mRNA abundances, ACO and ACS activities, and ACC content, causing lower ethylene evolution than the control fruit at the red stage (**Figures 3a-c**); moreover, except for *SlCTR1*, transcription of *SlETR3*/*4*/*7*, *SlCTR1*, *SlEIN2*, *SlEIL1*, *SlEBF1*/*2*, and *SlERF1* were downregulated as well (**Figure 3d**). Similar phenomenon was also in the MeJA-treated ‘Xiahui 8’ peach (Zhu et al., 2022).

Climacteric ethylene functions in the generation of many volatiles via regulating gene transcription, enzyme activities, and substrate generation in their biosynthesis pathways (Wang et al., 2016; Jia et al., 2023). For instance, *SlPSY1* expression level and thus the availability of carotenoids, which are associated with apocarotenoid volatile production (Cao et al., 2024; Tieman et al., 2006), is under the control of ethylene (Alba et al., 2005; Klee and Giovannoni, 2011); consistently, transient overexpression of *SlACS2* in ‘MicroTom’ tomato enhanced the ethylene evolution, upregulated *SlPSY1* mRNA level, promoted carotenoid generation, and thus elevated 6-methyl-5-hepten-2-one, geranyl acetone, and β-ionone formation (Jia et al., 2023). With the aid of *SlACO1*-transgenic fruit, the metabolism of the above-mentioned six key volatiles was under the control of ethylene (**Supplementary Figures S3**, **S4**; **Supplementary Table S5**). By considering the alteration of ethylene metabolism after exogenous MeJA treatment, our study suggested that the decrement of six key volatiles might be due to the mitigated ethylene biosynthesis & signaling transduction (**Figure 2**-**1**; **Supplementary Table S2**).

Until recently, several studies have explored that JA-Ile is the bioactive molecule in JAs signaling transduction (Fonseca et al., 2009); and MYC2 & MED25, two components in JAs signaling transduction process, positively regulate ethylene metabolism and thus fruit ripening (Deng et al., 2023; Li et al., 2017). Then, we assayed their alterations in the red fruit after MeJA fumigation at breaker stage. In this study, exogenous application of MeJA at breaker stage downregulated *SlLOXD*, *SlAOC*, *SlAOS*, *SlOPR3* expression levels, mitigated LOX, AOC, AOS, OPR activities, and thus suppressed endogenous JA-Ile formation in the red ‘FL 47’ tomato (**Figures 2a-c**); moreover, *SlMYC2* and *SlMED25* mRNA abundances were also inhibited as well (**Figure 2d**). Similar phenomenon was observed in ‘Xiahui 8’ peach after MeJA treatment (Zhu et al., 2022). By considering the occurrence of the peak of climacteric ethylene in ‘Xiahui 8’ peach (Zhu et al., 2022) and ‘FL 47’ tomato (Wang et al., 2019), these results implies that postharvest application of MeJA at early ripening stage might possess a negative impact on JA biosynthesis & signaling in the climacteric fruit at later ripening stage.

**Figure 6:**
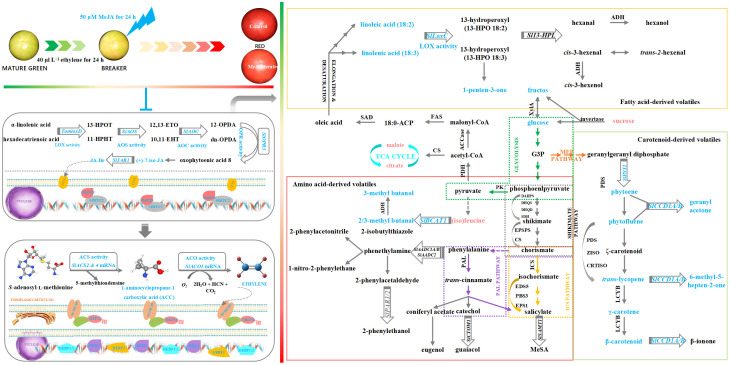
Schematic model on the impact of exogenous MeJA fumigation at breaker stage on aroma profile in the red ‘FL 47’ tomato. MeJA fumigation of breaker ‘FL 47’ tomato suppressed endogenous JAs (especially JA-Ile) and then ethylene biosynthesis & signaling transduction in the red fruit in association with the downregulated gene (*SlLOXD*, *SlAOC*, *SlAOS*, *SlOPR3*, *SlACO1*, *SlACS2/4*, *SlMYC2*, *SlMED25*, *SlETR3/4/7*, *SlEIN2*, *SlEIL1*, *SlEBF1/2*, and *SlERF1*) mRNA abundance, enzyme (LOX, AOC, AOS, OPR, ACO, and ACS) activities, and ACC content. Afterwards, the mitigated ethylene metabolism would in turn inhibit gene (*SlLOXC*, *SlPSY1*, *SlCCD1A/B*, and *SlBCAT1*) transcription and substrate (phytoene, phytofluene, *trans*-lycopene, γ-carotenoid, β-carotenoid, linoleic acid, and linolenic acid) production in their biosynthesis pathways, causing the reduced levels of six key volatiles (including 1-penten-3-one, geranyl acetone, 6-methyl-5-hepten-2-one, 2-methyl butanal, and 3-methyl butanal/ol). The above-mentioned outcome resulted in aroma profile change in the red fruit. Additionally, sugars and organic acids, including TSS, TA, glucose, fructose, sucrose, malate, and citrate were impacted by MeJA treatment as well, causing the alteration of taste profile. The schematic model integrates MeJA→ JA-Ile→ethylene→volatile modules inferred from the data (**Figures 1**–**4**). Blue (or red) color of words in the figure represented the negative (or positive) impact of MeJA treatment on gene expressions, enzyme activities, and metabolite production.

In combination, a schematic model on the impact of exogenous MeJA fumigation at breaker stage on aroma profile in the red ‘FL 47’ tomato was proposed as illustrated in **Figure 6**. MeJA fumigation of breaker ‘FL 47’ tomato suppressed endogenous JAs (especially JA-Ile) and then ethylene biosynthesis & signaling transduction in the red fruit in association with the downregulated gene (*SlLOXD*, *SlAOC*, *SlAOS*, *SlOPR3*, *SlACO1*, *SlACS2/4*, *SlMYC2*, *SlMED25*, *SlETR3/4/7*, *SlEIN2*, *SlEIL1*, *SlEBF1/2*, and *SlERF1*) mRNA abundance, enzyme (LOX, AOC, AOS, OPR, ACO, and ACS) activities, and ACC content. Afterwards, the mitigated ethylene metabolism would in turn inhibit gene (*SlLOXC*, *SlPSY1*, *SlCCD1A/B*, and *SlBCAT1*) transcription and substrate (phytoene, phytofluene, *trans*-lycopene, γ-carotenoid, β-carotenoid, linoleic acid, and linolenic acid) production in their biosynthesis pathways, causing the reduced levels of six key volatiles (including 1-penten-3-one, geranyl acetone, 6-methyl-5-hepten-2-one, 2-methyl butanal, and 3-methyl butanal/ol). The above-mentioned outcome resulted in aroma profile change in the red fruit. Additionally, sugars and organic acids, including TSS, TA, glucose, fructose, sucrose, malate, and citrate were impacted by MeJA treatment as well, causing the alteration of taste profile.

## 5 Conclusion

In this study, exogenous MeJA treatment at breaker stage considerably inhibited endogenous JAs (especially JA-Ile) and then ethylene biosynthesis & signaling transduction process in the red ‘FL 47’ tomato, which was associated with the reduced production of six key volatiles and the alteration of aroma profile. Consistently, gene (*SlLOXC*, *SlPSY1*, *SlCCD1A*/*B*, and *SlBCAT1*) expression profile as well as substrate (phytoene, phytofluene, *trans*-lycopene, γ-carotenoid, β-carotenoid, (iso)leucine, linoleic and linolenic acids) formation in their biosynthesis pathways were altered by MeJA fumigation as well. With the aid of transgenic technology, the metabolism of the above-mentioned key volatiles was under the control of ethylene, and endogenous JA-Ile content was positively associated with ethylene evolution in the ripening fruit. Therefore, our study suggested that inhibition of endogenous JAs (especially JA-Ile) and then ethylene metabolism by MeJA treatment might be responsible for volatile profile change in the red tomato.

## Data Availability

The datasets presented in this study can be found in online repositories. The names of the repository/repositories and accession number(s) can be found in the article/**Supplementary Material**.

## Supplementary material

Supplementary Figure 1 Schematic model on the experimental procedure in this study. **(a)** The experimental procedure. Uniform and defect-free ‘FL 47’ tomato at mature green stage were exposed to ethylene at 20°C to initiate and synchronize the ripening process; afterwards, the breaker fruit were selected and then divided into two lots for 50 µM MeJA and H_2_O (control) treatments based on the method of Wang et al. (2015), and post treatment ripening was at 20 °C. Fruit were sampled at the same time when the control and MeJA-treated fruits turned red. **(b)** Quality attributes (color, firmness, and weight loss). Data represent means ± standard deviation (SD) from three biological replicates; and significant differences (*p < 0.05; **p < 0.01; ***p < 0.001) were determined via one-way ANOVA followed by Tukey’s HSD post-hoc test.

Supplementary Figure 2 Impact of transient transformation of tomato with *SlMYC2* gene on key volatile production. **(a)** Transient overexpression of *SlMYC2* gene. For the generation of gene-overexpressing fruit, *SlMYC2* ORF without stop codon was amplified and inserted into the pCAMBIA1300 vector; afterwards, the recombinant vector was then transformed into *A. tumefaciens* strain GV3101 before injection into mature green ‘MicroTom’ tomato through the carpopodium tissue; fruit infiltrated with empty pCAMBIA1300 vector were used as the control. Pericarp tissue was sampled at the same sampling time when the control and SlMYC2-overexpressed fruits turned red. **(b)** Transient silence of SlMYC2 gene. For the generation of gene-silenced fruit, about 250-bp fragment of SlMYC2 ORF was amplified from ‘FL 47’ tomato and inserted into pTRV2 vector; afterwards, the constructed plasmid and pTRV1 were transformed into *A. tumefaciens* strain GV3101, respectively, and then combined in a ratio of 1:1 before injection into mature green ‘MicroTom’ tomato through the carpopodium tissue; fruit infiltrated with empty pTRV2 and pTRV1 vectors were used as the control. Pericarp tissue was sampled at the same time when the control and SlMYC2-silenced fruits turned red. Data represents the mean value of three biological replicates. Color scale represents normalized log_2_-transformed (mean value of volatile abundance + 1), where red indicates a high level, grey indicates a low level, and white indicates a medium level.

Supplementary Figure 3 Impact of transient overexpression of *SlACO1* gene in tomato on key volatile production. **(a)** Visual quality. **(b)***SlACO1* expression level and ethylene evolution. **(c)** Substrate abundance. **(d)** Gene expression profile. **(e)** Key volatile production. *SlACO1* ORF without stop codon was amplified and inserted into the pCAMBIA1300 vector; afterwards, the recombinant vector was then transformed into *A. tumefaciens* strain GV3101 before injection into mature green ‘MicroTom’ tomato through the carpopodium tissue. Fruit infiltrated with empty pCAMBIA1300 vector were used as the control. Pericarp tissue was sampled at the same sampling time when the control and *SlACO1*-overexpressed fruits turned red. The expression level of each gene in the control fruit was set as 1.0 based on qRT-PCR result. Data represent means ± standard deviation (SD) from three biological replicates; and significant differences (*p < 0.05; **p < 0.01; ***p < 0.001) were determined via one-way ANOVA followed by Tukey’s HSD post-hoc test. Color scale represents normalized log_2_-transformed (mean value of volatile abundance + 1), where red indicates a high level, grey indicates a low level, and white indicates a medium level.

Supplementary Figure 4 Impact of transient silence of *SlACO1* gene in tomato on key volatile production. **(a)** Visual quality. **(b)***SlACO1* expression level and ethylene evolution. **(c)** Substrate abundance. **(d)** Gene expression profile. **(e)** Key volatile production. About 250-bp fragment of *SlACO1* ORF was amplified from ‘FL 47’ tomato and inserted into pTRV2 vector; afterwards, the constructed plasmid and pTRV1 were transformed into *A. tumefaciens* strain GV3101, respectively, and then combined in a ratio of 1:1 before injection into mature green ‘MicroTom’ tomato through the carpopodium tissue. Fruit infiltrated with empty pTRV2 and pTRV1 vectors were used as the control. Pericarp tissue was sampled at the same time when the control and *SlACO1*-silenced fruits turned red. The expression level of each gene in the control fruit was set as 1.0 based on qRT-PCR result. Data represent means ± standard deviation (SD) from three biological replicates; and significant differences (*p < 0.05; **p < 0.01; ***p < 0.001) were determined via one-way ANOVA followed by Tukey’s HSD post-hoc test. Color scale represents normalized log_2_-transformed (mean value of volatile abundance + 1), where red indicates a high level, grey indicates a low level, and white indicates a medium level.
