# Supplementary figures and images for: Exogenous MeJA modulates postharvest tomato aroma by suppressing JAs-ethylene signaling crosstalk
Source: Front Plant Sci. 2025 Dec 1;16:1712703. doi: 10.3389/fpls.2025.1712703 (PMC12703787; doi:10.3389/fpls.2025.1712703)

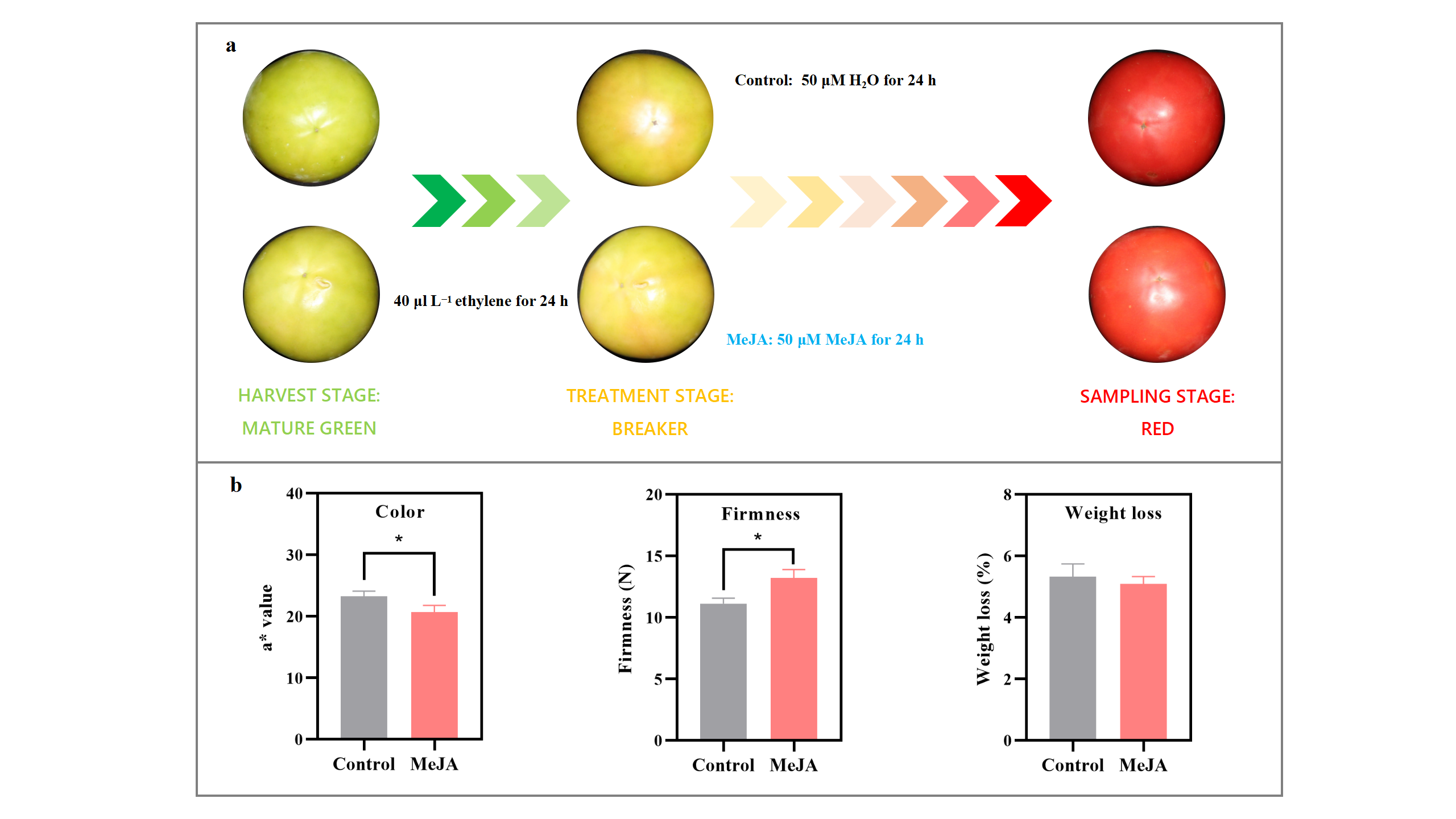

Supplement: Supplementary Figure 1 [file Image1.tif]

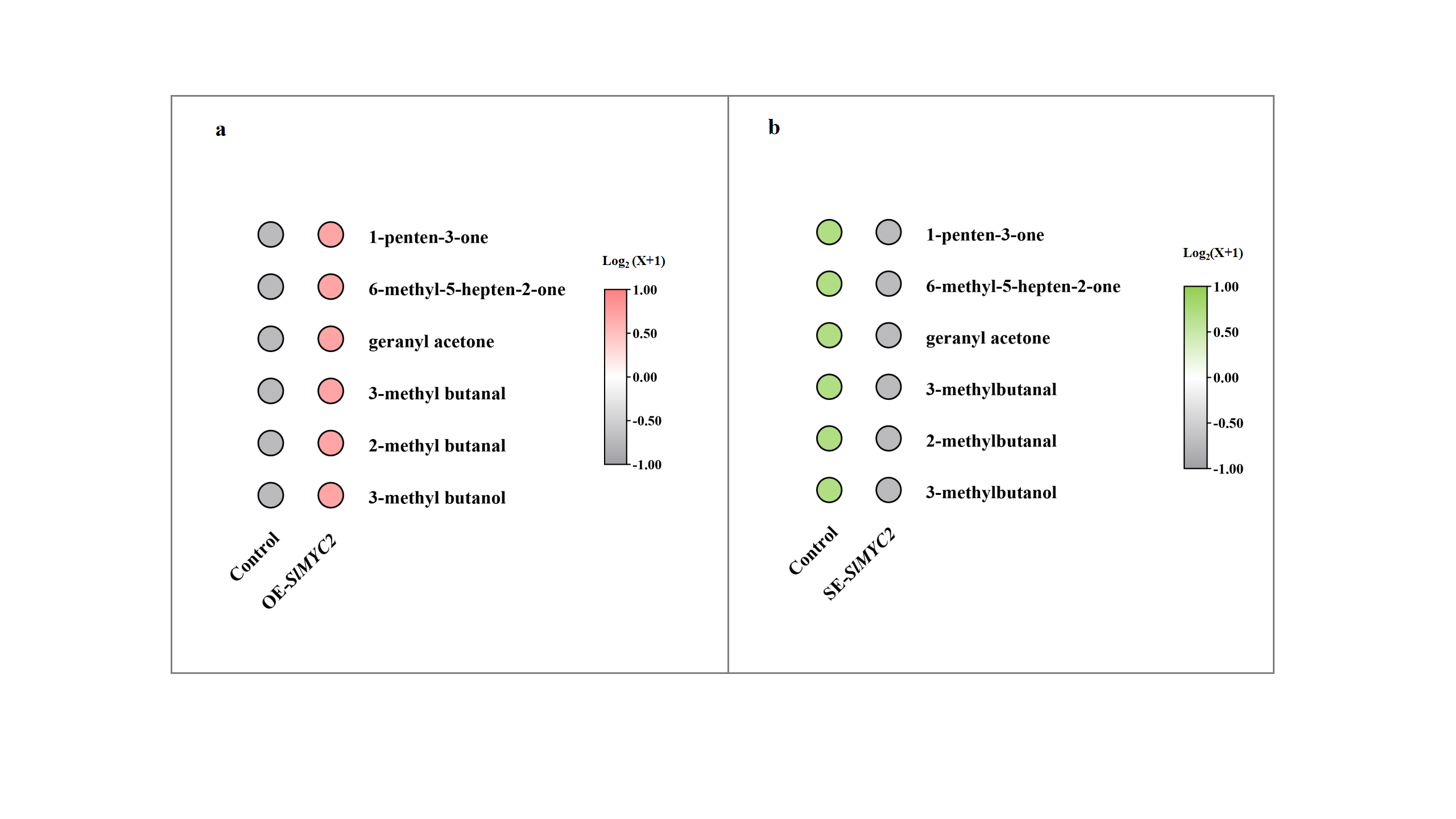

Supplement: Supplementary Figure 2 [file Image2.tif]

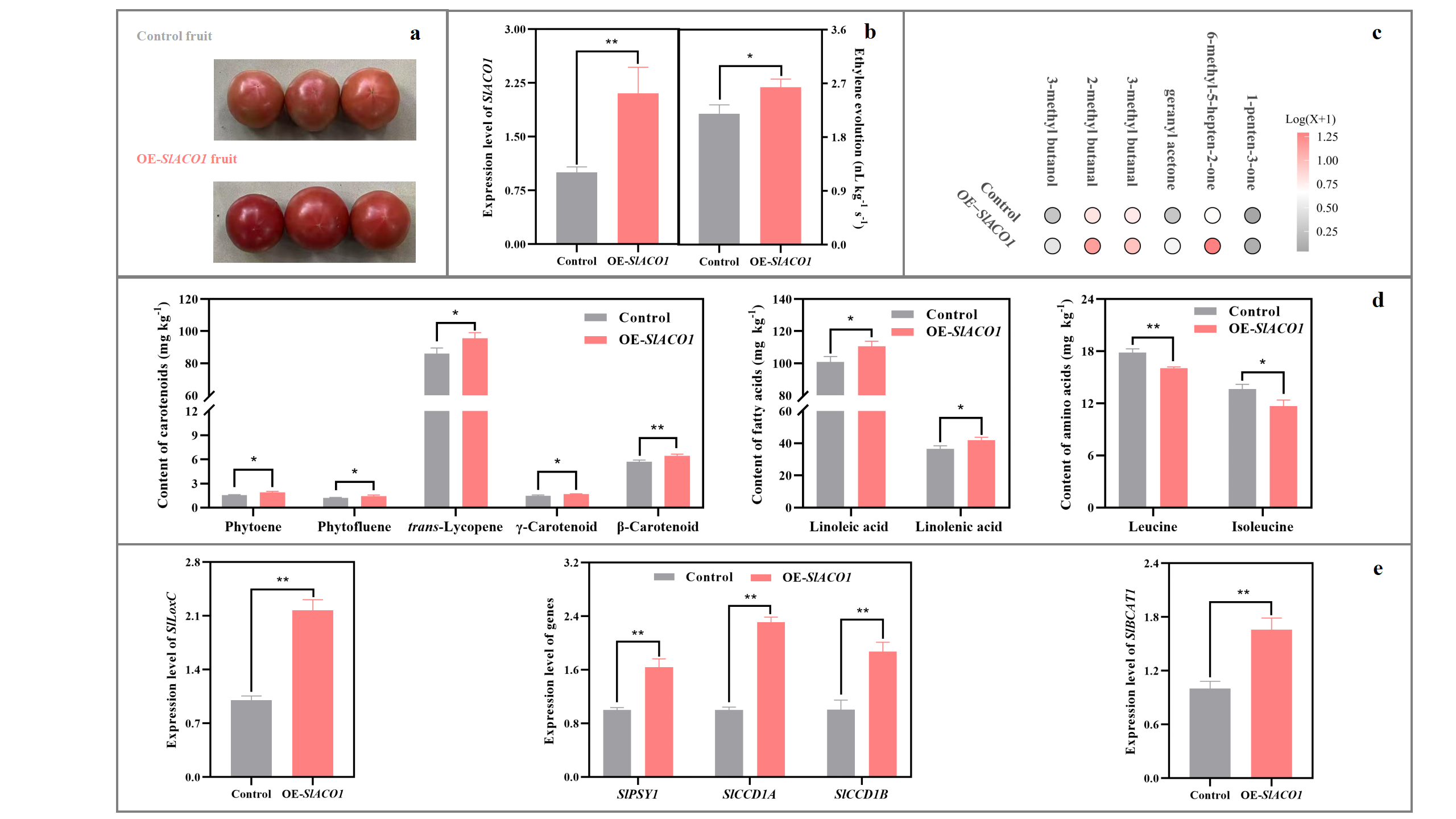

Supplement: Supplementary Figure 3 [file Image3.tif]

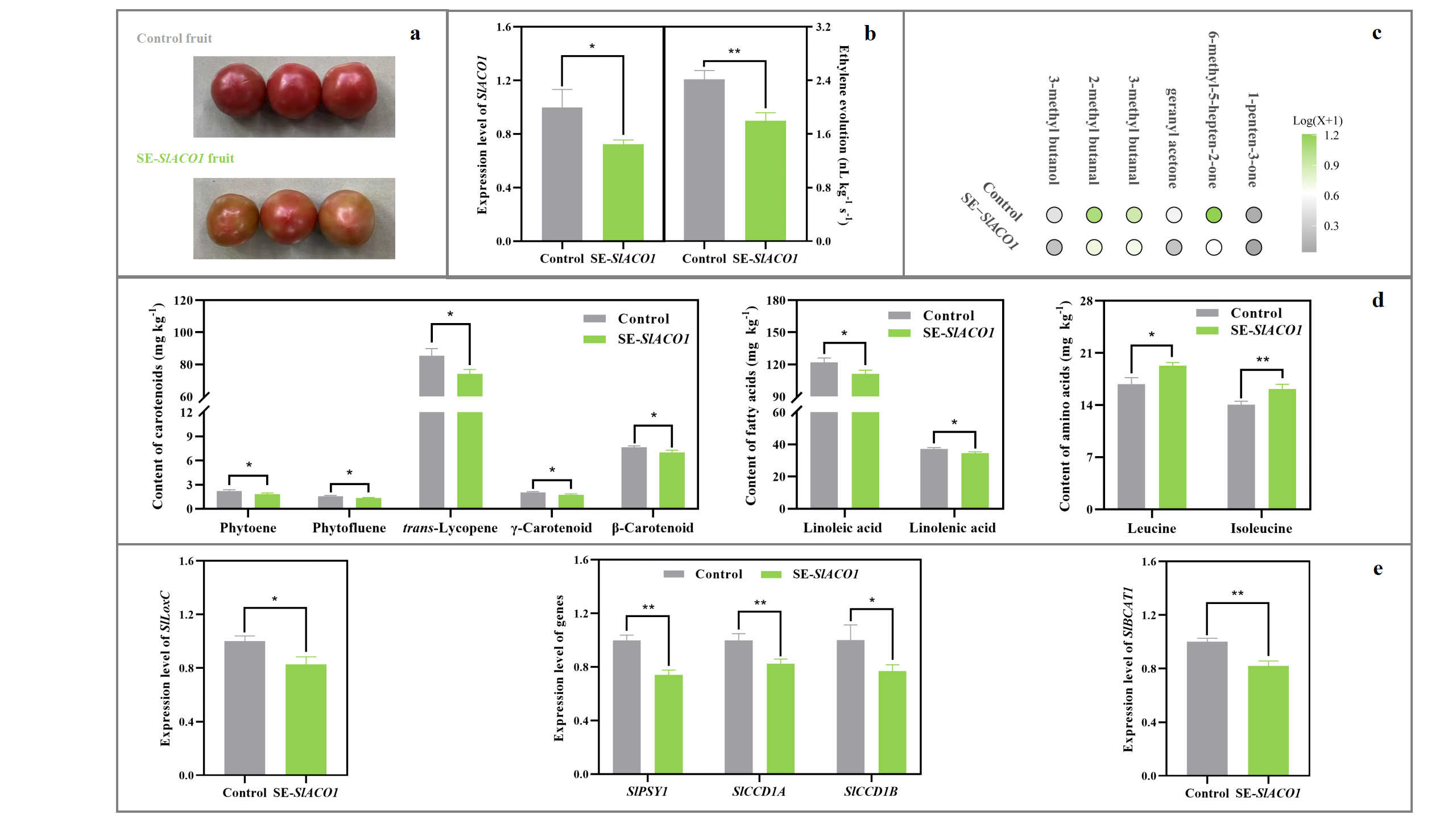

Supplement: Supplementary Figure 4 [file Image4.tif]
