# Supplementary material for: Exogenous MeJA modulates postharvest tomato aroma by suppressing JAs-ethylene signaling crosstalk
Source: Front Plant Sci. 2025 Dec 1;16:1712703. doi: 10.3389/fpls.2025.1712703 (PMC12703787; doi:10.3389/fpls.2025.1712703)
Supplement: Supplementary file 5 [file DataSheet1.docx]

**Table S1 Primers used in this study**

| **Gene name** | | | **Gene ID** | **Purpose/sequence** | | | |
| --- | --- | --- | --- | --- | --- | --- | --- |
|  |  |  |  | **Forward sequence** | | **Reverse sequence** | |
| **RT-qPCR** | |  |  |  |  |  |  |
|  | **Ethylene biosynthesis** | |  |  |  |  |  |
|  |  | *SlACS2* | Solyc01g095080 | F | CTACGCAGCCACTGTCTTTGAC | R | TGATTCCGACTCTAAATCCTGGTAA |
|  |  | *SlACS4* | Solyc05g050010 | F | TTGCGACGAAATATATGCTGCT | R | CACTCGAAATCCTGGAAAACCT |
|  |  | *SlACO1* | Solyc07g049530 | F | ACTATCCACCATGTCCTAAGCCCG | R | TCTGTTTGTGCAATTACTCTGTGCAGC |
|  | **Ethylene signaling transduction** | | |  |  |  |  |
|  |  | *SlETR3* | Solyc09g075440 | F | GGCATCTTAAGAGAGGCCGTTA | R | TAAATCCAGAGCCAAAGCAAGAG |
|  |  | *SlETR4* | Solyc06g053710 | F | ATCCTCATTCCAAATCGTCCT | R | TTTCATCAGCAGTCGCAGTTA |
|  |  | *SlETR7* | Solyc05g055070 | F | GGGTATGTTGGATCTTGAGGCA | R | GGCGGTTAGGATTTTCGCAAC |
|  |  | *SlCTR1* | Solyc10g083610 | F | GCATATCCCCTAGTTGCATCAC | R | CATGGAAACCAGTTCCTCTTCT |
|  |  | *SlEIN2* | Solyc09g007870 | F | GTCCGGGGTCATACAAAAGTCT | R | GAGCTTCTGTTAACTGCCTCCT |
|  |  | *SlEIL1* | Solyc06g073720 | F | TTTGGCTCGAGAACTCTATC | R | ATGAGATTGTTGCTGAAGAG |
|  |  | *SlEBF1* | Solyc12g009560 | F | ATGTGATGGATACCTTACCAG | R | CCGACATTAGTAATACCACGA |
|  |  | *SlEBF2* | Solyc08g060810 | F | ATTGCCATCACTGACATAGC | R | AGTTATAGCAAGCGACCTC |
|  |  | *SlERF1* | Solyc05g051200 | F | GACAGAGACATTGGGGTTCTTG | R | TTAGCCTTGCTGCTTCATCATA |
|  | **JAs biosynthesis** | |  |  |  |  |  |
|  |  | *SlLOXD* | Solyc03g122340 | F | TGTGCCACTGGTAACTGGAT | R | TCCAAGCTTGCATGTGTACG |
|  |  | *SlAOC* | Solyc02g085730 | F | CCGTTCAGGGAGCGTACTTA | R | ACCGCCGTACACAACAATTC |
|  |  | *SlAOS* | Solyc04g079730 | F | GAACCTCCGGTAGCATCACA | R | TTCTTCTCCGATGAACCGAT |
|  |  | *SlOPR3* | Solyc07g007870 | F | ATAGGAGCTGATCGCGTAGG | R | TAGGCAAGCTTGGAACCAGA |
|  | **JAs signaling transduction** | | |  |  |  |  |
|  |  | *SlMYC2* | Solyc08g076930 | F | AGCAGGAGCATCGGAAGAA | R | CCAAATCGGGCTGGAACTA |
|  |  | *SlMED25* | Solyc12g070100 | F | CGTCTGTTCGTCCTGCTCTT | R | GCCTAGAACTTGGTGTGCCT |
|  |  | *SlMTB1* | Solyc01g096050 | F | TGCTGTTAGGAAAGCGGAGG | R | TGGGGTTGGAGGAGCATAGA |
|  | **Carotenoid-derived volatile biosynthesis** | | |  |  |  |  |
|  |  | *SlPSY1* | Solyc03g031860 | F | AACTTGTTGATGGCCCAAAC | R | CTGTATC GGACAAAGCACCA |
|  |  | *SlCCD1A* | Solyc01g087250 | F | TTGATTACCTGCCGCCTTGT | R | CATATAGCTCATTGCAGAAATTC |
|  |  | *SlCCD1B* | Solyc01g087260 | F | AGAATCCAGATCTTGACGCGATT | R | CCTCATCTCATACAACTCATTTGT |
|  | **Fatty acid-derived volatile biosynthesis** | | |  |  |  |  |
|  |  | *SlLoxC* | Solyc01g006540 | F | AAATGAGCACCACAAGGAAATGTAT | R | ACCCAAGAGTTGCATGTAATTTCA |
|  | **Branched-chain volatile biosynthesis** | | |  |  |  |  |
|  |  | *SlBCAT1* | Solyc12g088220 | F | AGGGCTCTATTTACTTCTTTTGAG | F | CATACACATTCTTTTAGCACCAATT |
|  |  | *SlPAR1* | Solyc01g008530 | F | CGGGTCATGTGCCAAAGC | R | GGCTGACCACTGTAAGCAACTG |
|  |  | *SlPAR2* | Solyc01g008550 | F | ATGATCAAAAAAAGGTAGATCACTTGAC | R | CCAGTAGGTCCGCTTTGAACA |
|  | **Housekeeping gene** | |  |  |  |  |  |
|  |  | *SlActin* | Solyc03g078400 | F | GTGCGAGTGTCCTGTCTGTT | R | CCAAGGGATGGTGTAGCCAG |
|  |  | *SlGAPDH* | Solyc05g014470 | F | GGCTGCAATCAAGGAG GAA | R | AAATCAATCACACGGG AACTG |
| **Gene function validation** | | |  |  |  |  |  |
|  | **Gene overexpression** | |  |  |  |  |  |
|  |  | *SlACO1* | Solyc01g095080 | F | gagaacacgggggactctagaATGGAGAACTTCCCAATTATTAAC | R | cccttgctcaccatggatccCTAAGCACTTGCAATTGGATCACT |
|  |  | *SlLOXD* | Solyc03g122340 | F | gagaacacgggggactctagaATGGCACTTGCTAAAGAAATTATG | R | cccttgctcaccatggatccTCATATCGATACACTATTTGGAACACCCC |
|  |  | *SlMYC2* | Solyc08g076930 | F | gagaacacgggggactctagaATGACTGAATACAGCTTGCCCACC | R | cccttgctcaccatggatccTTAGTGTGTTTCAGCAATTTTCG |
|  |  | *SlMTB1* | Solyc01g096050 | F | gagaacacgggggactctagaATGGTTACTGGGAATATGTTGTG | R | cccttgctcaccatggatccTTATTGCCCTACCGGTGAAAGTTGCC |
|  | **Gene silence** | |  |  |  |  |  |
|  |  | *SlACO1* | Solyc01g095080 | F | gtgagtaaggttaccgaattcACTATCCACCATGTCCTAAGCCCG | R | gtgagtaaggttaccgaattcTCTGTTTGTGCAATTACTCTGTGCAGC |
|  |  | *SlLOXD* | Solyc03g122340 | F | gtgagtaaggttaccgaattcTGTGCCACTGGTAACTGGAT | R | gtgagtaaggttaccgaattcTCCAAGCTTGCATGTGTACG |
|  |  | *SlMYC2* | Solyc08g076930 | F | gtgagtaaggttaccgaattcAGCAGGAGCATCGGAAGAA | R | gtgagtaaggttaccgaattcCCAAATCGGGCTGGAACTA |
|  |  | *SlMTB1* | Solyc01g096050 | F | gtgagtaaggttaccgaattcTGCTGTTAGGAAAGCGGAGG | R | gtgagtaaggttaccgaattcTGGGGTTGGAGGAGCATAGA |

**Table S2 Impact of exogenous MeJA fumigation at breaker stage on volatile profile in the red ‘FL 47’ tomato ^a^**

| **Volatile compounds** | | **Retention index (RI)** | **Odor description^b^** | **Odor threshold in water (mg·kg^-1^)^c^** | **Treatments/Concentration (mg·kg^-1^)** | |
| --- | --- | --- | --- | --- | --- | --- |
|  |  |  |  |  | **Control** | **MeJA** |
| **Aldehydes** | |  |  |  |  |  |
|  | butanal | 0579 | Pungent, green | 0.0090 | 0.0163 a^e^ | 0.0084 b |
|  | **3-methyl butanal ^d^** | **0636** | **Malt** | **0.00015-0.00020** | **0.78 a** | **0.44 b** |
|  | **2-methyl butanal ^d^** | **0645** | **Cocoa, almond, malt** | **0.0030** | **0.63 a** | **0.32 b** |
|  | 2-methyl-2-butenal | 0725 | Green, fruit | 0.50 | 0.080 a | 0.054 b |
|  | *trans*-2-pentenal ^d^ | 0741 | Strawberry, fruit, tomato | 1.50 | 0.025 a | 0.026 a |
|  | ***cis*-3-hexenal ^d^** | **0782** | **Leaf, green** | **0.00025** | **3.12 a** | **3.89 a** |
|  | **hexanal ^d^** | **0785** | **Grass, tallow, fat** | **0.0045-0.005** | **2.15 a** | **2.70 a** |
|  | ***trans*-2-hexenal ^d^** | **0841** | **Green, leaf** | **0.017** | **0.70 a** | **0.81 a** |
|  | heptanal | 0888 | Fat, citrus, rancid | 0.0030 | 0.0053 a | 0.0044 b |
|  | *trans*-2, *trans*-4-hexadienal | 0900 | Green | 0.060 | 0.74 b | 1.19 a |
|  | benzaldehyde | 0958 | Almond, burnt sugar | 0.35 | 0.021 a | 0.018 a |
|  | **2-phenylacetaldehyde ^d^** | **1029** | **Hawthorne,honey, sweet** | **0.0040** | **1.36 a** | **1.44 a** |
|  | nonanal | 1071 | Fat, citrus, green | 0.0010 | 0.00069 a | 0.00144 a |
|  | β-cyclocitral | 1182 | Mint | 0.0050 | 0.00042 a | 0.00044 a |
|  | neral | 1196 | Lemon | 0.030 | 0.0115 a | 0.0073 b |
|  | geranial | 1224 | Lemon, mint | 0.032 | 0.036 a | 0.021 b |
| **Alcohols** | |  |  |  |  |  |
|  | **3-methyl butanol ^d^** | **0713** | **Whiskey, malt, burnt** | **0.25-0.30** | **0.38 a** | **0.23 b** |
|  | 2-methyl butanol | 0717 | Malt, wine, onion | 0.25-0.30 | 0.35 a | 0.20 b |
|  | 4-methyl pentanol | 0823 | Pungent | 0.82-4.10 | 0.0169 a | 0.0067 b |
|  | 3-methyl pentanol | 0832 | Pungent | 0.83-4.10 | 0.024 a | 0.010 b |
|  | 6-methyl-5-hepten-2-ol | 0975 | Mushroom, musty, mouldy, earthy | 2.00 | 0.0121 a | 0.0055 b |
|  | 2-ethyl hexanol | 0992 | Rose, green | 0.83-1.50 | 0.013 a | 0.013 a |
|  | linalool | 1066 | Flower, lavender | 0.0060 | 0.0087 a | 0.0079 a |
|  | **2-phenylethanol ^d^** | **1098** | **Honey, spice, rose, lilac** | **1.00-1.10** | **1.09 a** | **1.17 a** |
| **Hydrocarbons** | |  |  |  |  |  |
|  | nonane | 0882 | Alkane | 10.00 | 0.00042 a | 0.00037 a |
|  | α-pinene | 0925 | Pine, turpentine | 0.0060 | 0.000095 a | 0.000095 a |
|  | p-cymene | 1007 | Solvent, gasoline, citrus | 0.15 | 0.00026 a | 0.00024 a |
|  | d-limonene | 1011 | Lemon, orange | 0.010 | 0.062 a | 0.059 a |
|  | undecane | 1062 | Alkane | 10.00 | 0.0143 a | 0.0074 b |
|  | dodecane | 1145 | Alkane | 10.00 | 0.0020 a | 0.0010 b |
| **Ketones** | |  |  |  |  |  |
|  | 2-butanone | 0581 | Sweet | 7.00 | 0.107 a | 0.066 b |
|  | **1-penten-3-one ^d^** | **0666** | **Fruity, floral, green** | **0.0015** | **0.082 a** | **0.035 b** |
|  | **6-methyl-5-hepten-2-one ^d^** | **0963** | **Fruity, floral** | **0.050** | **0.40 a** | **0.27 b** |
|  | **geranyl acetone ^d^** | **1380** | **Sweet, floral, estery** | **0.060** | **0.86 a** | **0.56 b** |
| **Esters** | |  |  |  |  |  |
|  | butyl acetate | 0753 | Pear | 0.066 | 0.0078 a | 0.0071 a |
|  | 2-methylbutyl acetate | 0860 | Fruit | 0.0050-0.0110 | 0.0051 a | 0.0037 b |
| **Oxygen-containing heterocyclic compounds** | | |  |  |  |  |
|  | 2-methyl furan | 0593 | Chocolate | 3.50-4.00 | 0.055 a | 0.036 b |
|  | 2-ethyl furan | 0678 | Rum, coffee and chocolate | - | 0.024 a | 0.025 a |
|  | 5-ethyl-2(5H)-furanone | 0946 | Caramellic | - | 1.01 a | 1.05 a |
| **Sulfur- and nitrogen- containing heterocyclic compounds** | | | |  |  |  |
|  | **2-isobutylthiazole ^d^** | **1017** | **Tomato leaf, green** | **0.0035** | **0.0072 a** | **0.0077 a** |
|  |  |  |  |  |  |  |
| **Total concentration by chemical class** | | |  |  |  |  |
|  | **Aldehydes** |  |  |  | **9.73 b** | **10.97 a** |
|  | **Alcohols** |  |  |  | **1.89 a** | **1.64 a** |
|  | **Hydrocarbons** |  |  |  | **0.079 a** | **0.068 a** |
|  | **Ketones** |  |  |  | **1.45 a** | **0.93 b** |
|  | **Esters** |  |  |  | **0.013 a** | **0.011 a** |
|  | **Oxygen-containing heterocyclic compounds** |  |  |  | **1.09 a** | **1.11 a** |
|  | **Sulfur- and nitrogen- containing heterocyclic compounds** |  |  |  | **0.0072 a** | **0.0077 a** |
| **Total volatiles** | |  |  |  | **14.26 a** | **14.73 a** |
| ^a^ ‘FL 47’ tomato were treated with MeJA or H_2_O (control) for 24 h prior to ripening at 20 ℃; fruit were sampled when the MeJA-treated fruit turned red. | | | | | | |
| ^b^ Odor descriptions of 6-methyl-5-hepten-2-one and geranyl acetone were adapted from Klee (2010), while others from Acree and Arn (2010). | | | | | | |
| ^[c](file:///C:\\Users\\DELL\\Desktop\\小论文\\New-20250725-番茄文章\\New-20250725-番茄文章\\New-20250718-番茄文章\\2025-07-18-S-Table.xlsx#RANGE!_ENREF_57)^ [Odor threshold values (detection) in water adapted from van Gemert (2003).](file:///C:\\Users\\DELL\\Desktop\\小论文\\New-20250725-番茄文章\\New-20250725-番茄文章\\New-20250718-番茄文章\\2025-07-18-S-Table.xlsx#RANGE!_ENREF_57) | | | | | | |
| ^d^ Key tomato flavor compounds recommended by Klee (2010). | | | | | | |
| ^e^ Mean values that are not followed by the same letter within the same row show significant difference using Tukey’s Honest Significant Difference (HSD) post-hoc test (*p* < 0.05). | | | | | | |

**Table S3 Effect size summary for six key MeJA-responsive volatiles**

| **Compound** | **Mean (Control)± SD** | **Mean (MeJA) ± SD** | **Cohen's d** | **95% CI** | **Interpretation** |
| --- | --- | --- | --- | --- | --- |
| 1-penten-3-one | 0.083 ± 0.015 | 0.037 ± 0.015 | 3.06 | [0.41, 5.59] | Large |
| 2-methyl butanal | 0.63 ± 0.038 | 0.32 ± 0.091 | 4.46 | [1.06, 7.79] | Large |
| 3-methyl-1-butanol | 0.38 ± 0.055 | 0.23 ± 0.042 | 3.07 | [0.42, 5.61] | Large |
| 3-methyl butanal | 0.79 ± 0.076 | 0.44 ± 0.049 | 5.48 | [1.49, 9.43] | Large |
| 6-methyl-5-hepten-2-one | 0.40 ± 0.044 | 0.27 ± 0.042 | 3.13 | [0.45, 5.70] | Large |
| geranyl acetone | 0.87 ± 0.090 | 0.56 ± 0.067 | 3.91 | [0.82, 6.92] | Large |

Note: Mean ± SD values are shown for control and MeJA-treated fruit (n = 3 per treatment). Effect sizes are calculated as Cohen's d, with 95 % confidence

intervals estimated by bootstrap resampling (10,000–20,000 draws) from the observed replicates. All six compounds exhibit large standardized treatment effects.

**Table S4 Impact of transient transformation of tomato with *SlMYC2* gene on key volatile production ^a^**

| **Volatile compounds** | **Retention index (RI)** | **Odor description ^b^** | **Odor threshold in water (mg·kg^-1^)^c^** | | **Treatments/Concentration (mg·kg^-1^)** | | | | | |
| --- | --- | --- | --- | --- | --- | --- | --- | --- | --- | --- |
|  |  |  |  |  | **Overexpression** | | **Silence** | | | |
|  |  |  |  |  | **Control(empty pCAMBIA1300 vector)** | **OE*-SlMYC2 (SlMYC2*::pCAMBIA1300 vector)** | **Control (pTRV1 & pTRV2)** | | | **SE-*SlMYC2* (pTRV1**  **& pTRV2::*SlMYC2)*** |
| 1-penten-3-one | 0666 | Fruity, floral, green | 0.0015 | | 0.038 b ^d^ | 0.055 a | 0.040 a ^d^ | | | 0.025 b |
| 6-methyl-5-hepten-2-one | 0963 | Fruity, floral | 0.050 | | 0.72 b | 1.28 a | 0.92 a | | | 0.70 b |
| geranyl acetone | 1380 | Sweet, floral, estery | 0.060 | | 0.25 b | 0.40 a | 0.38 a | | | 0.25 b |
| 3-methyl butanal | 0636 | Malt | 0.00015-0.00020 | | 0.80 b | 1.05 a | 0.64 a | | | 0.44 b |
| 2-methyl butanal | 0645 | Cocoa, almond, malt | 0.0030 | | 1.03 b | 1.32 a | 0.84 a | | | 0.62 b |
| 3-methyl butanol | 0713 | Whiskey, malt, burnt | 0.25-0.30 | | 0.24 b | 0.36 a | 0.27 a | | | 0.20 b |
| ^a^ For the generation of gene-overexpressing fruit, *SlMYC2* ORF without stop codon was amplified and inserted into the pCAMBIA1300 vector; afterwards, the recombinant vector was then transformed into *A. tumefaciens* strain GV3101 before injection into mature green ‘MicroTom’ tomato through the carpopodium tissue; fruit infiltrated with empty pCAMBIA1300 vector were used as the control. For the generation of gene-silenced fruit, about 250-bp fragment of *SlMYC2* ORF was amplified from ‘FL 47’ tomato and inserted into pTRV2 vector; afterwards, the constructed plasmid and pTRV1 were transformed into *A. tumefaciens* strain GV3101, respectively, and then combined in a ratio of 1:1 before injection into mature green ‘MicroTom’ tomato through the carpopodium tissue; fruit infiltrated with empty pTRV2 and pTRV1 vectors were used as the control. | | | | | | | | | | |
| ^b^ Odor descriptions of 6-methyl-5-hepten-2-one and geranyl acetone were adapted from Klee (2010), while others from Acree and Arn (2010). | | | | | | |  |  |  | |
| ^[c](file:///C:\\Users\\DELL\\Desktop\\小论文\\New-20250725-番茄文章\\New-20250725-番茄文章\\New-20250718-番茄文章\\2025-07-18-S-Table.xlsx#RANGE!_ENREF_57)^ [Odor threshold values (detection) in water adapted from van Gemert (2003).](file:///C:\\Users\\DELL\\Desktop\\小论文\\New-20250725-番茄文章\\New-20250725-番茄文章\\New-20250718-番茄文章\\2025-07-18-S-Table.xlsx#RANGE!_ENREF_57) | | |  |  | |  |  |  |  | |
| ^d^ Mean values that are not followed by the same letter within the same row show significant difference using Tukey’s Honest Significant Difference (HSD) post-hoc test (*p* < 0.05). | | |  |  | |  |  |  |  | |

**Table S5 Impact of transient transformation of tomato with *SlACO1* gene on key volatile production ^a^**

| **Volatile compounds** | **Retention index (RI)** | **Odor description ^b^** | **Odor threshold in water (mg·kg^-1^) ^c^** | | **Treatments/Concentration (mg·kg^-1^)** | | | | | |
| --- | --- | --- | --- | --- | --- | --- | --- | --- | --- | --- |
|  |  |  |  |  | **Overexpression** | | **Silence** | | | |
|  |  |  |  |  | **Control (empty pCAMBIA1300 vector)** | **OE*-SlACO1 (SlACO1*::pCAMBIA1300 vector)** | **Control (pTRV1 & pTRV2)** | | | **SE-*SlACO1* (pTRV1 & pTRV2::*SlACO1)*** |
| 3-methyl butanal | 0636 | Malt | 0.00015-0.00020 | | 0.70 b ^d^ | 0.97 a | 0.87 a ^d^ | | | 0.62 b |
| 2-methyl butanal | 0645 | Cocoa, almond, malt | 0.0030 | | 0.74 b | 1.22 a | 1.10 a | | | 0.66 b |
| 3-methyl butanol | 0713 | Whiskey, malt, burnt | 0.25-0.30 | | 0.19 b | 0.41 a | 0.35 a | | | 0.17 b |
| 1-penten-3-one | 0666 | Fruity, floral, green | 0.0015 | | 0.031 b | 0.071 a | 0.062 a | | | 0.027 b |
| 6-methyl-5-hepten-2-one | 0963 | Fruity, floral | 0.050 | | 0.60 b | 1.45 a | 1.31 a | | | 0.53 b |
| geranyl acetone | 1380 | Sweet, floral, estery | 0.060 | | 0.20 b | 0.52 a | 0.47 a | | | 0.18 b |
| ^a^ For the generation of gene-overexpressing fruit, *SlACO1* ORF without stop codon was amplified and inserted into the pCAMBIA1300 vector; afterwards, the recombinant vector was then transformed into *A. tumefaciens* strain GV3101 before injection into mature green ‘MicroTom’ tomato through the carpopodium tissue; fruit infiltrated with empty pCAMBIA1300 vector were used as the control. For the generation of gene-silenced fruit, about 250-bp fragment of *SlACO1* ORF was amplified from ‘FL 47’ tomato and inserted into pTRV2 vector; afterwards, the constructed plasmid and pTRV1 were transformed into *A. tumefaciens* strain GV3101, respectively, and then combined in a ratio of 1:1 before injection into mature green ‘MicroTom’ tomato through the carpopodium tissue; fruit infiltrated with empty pTRV2 and pTRV1 vectors were used as the control. | | | | | | | | | | |
| ^b^ Odor descriptions of 6-methyl-5-hepten-2-one and geranyl acetone were adapted from Klee (2010), while others from Acree and Arn (2010). | | | | | | |  |  |  | |
| ^[c](file:///C:\\Users\\DELL\\Desktop\\小论文\\New-20250725-番茄文章\\New-20250725-番茄文章\\New-20250718-番茄文章\\2025-07-18-S-Table.xlsx#RANGE!_ENREF_57)^ [Odor threshold values (detection) in water adapted from van Gemert (2003).](file:///C:\\Users\\DELL\\Desktop\\小论文\\New-20250725-番茄文章\\New-20250725-番茄文章\\New-20250718-番茄文章\\2025-07-18-S-Table.xlsx#RANGE!_ENREF_57) | | |  |  | |  |  |  |  | |
| ^d^ Mean values that are not followed by the same letter within the same row show significant difference using Tukey’s Honest Significant Difference (HSD) post-hoc test (*p* < 0.05). | | | | | | | |  |  | |
